# Thyroid hormone plus dual-specificity phosphatase-5 siRNA increases the number of cardiac muscle cells and improves left ventricular contractile function in chronic doxorubicin-injured hearts

**DOI:** 10.7150/thno.57456

**Published:** 2021-03-04

**Authors:** Lin Tan, Nikolay Bogush, Emmen Naqvi, John W. Calvert, Robert M. Graham, W. Robert Taylor, Nawazish Naqvi, Ahsan Husain

**Affiliations:** 1 Department of Medicine (Cardiology), Emory University School of Medicine, Atlanta, GA, USA.; 2 Department of Surgery, Carlyle Fraser Heart Center, Emory University School of Medicine, Atlanta, GA, USA.; 3 Victor Chang Cardiac Research Institute, Darlinghurst, New South Wales, Australia.; 4 Atlanta Veterans Affairs Medical Center, Cardiology Division, Atlanta, GA, USA.; 5 Emory University School of Medicine and Georgia Institute of Technology, Department of Biomedical Engineering, Atlanta, GA, USA.

**Keywords:** doxorubicin cardiotoxicity, heart failure, cardiomyocytes, DUSP5, thyroid hormone

## Abstract

**Rationale:** Doxorubicin is a widely used anticancer drug. However, its major side effect, cardiotoxicity, results from cardiomyocyte loss that causes left ventricle (LV) wall thinning, chronic LV dysfunction and heart failure. Cardiomyocyte number expansion by thyroid hormone (T3) during preadolescence is suppressed by the developmental induction of an ERK1/2-specific dual specificity phosphatase 5 (DUSP5). Here, we sought to determine if a brief course of combined DUSP5 suppression plus T3 therapy replaces cardiomyocytes lost due to preexisting doxorubicin injury and reverses heart failure.

**Methods:** We used *in vivo*-jetPEI to deliver DUSP5 or scrambled siRNA to ~5-week-old C57BL6 mice followed by 5 daily injections of T3 (2 ng/µg body weight). Genetic lineage tracing using *Myh6*-Mer*Cre*Mer::Rosa26fs-Confetti mice and direct cardiomyocyte number counting, along with cell cycle inhibition (danusertib), was used to test if this treatment leads to *de novo* cardiomyocyte generation and improves LV contractile function. Three doses of doxorubicin (20 µg/g) given at 2-weekly intervals, starting at 5-weeks of age in C57BL6 mice, caused severe heart failure, as evident by a decrease in LV ejection fraction. Mice with an ~40 percentage point decrease in LVEF post-doxorubicin injury were randomized to receive either DUSP5 siRNA plus T3, or scrambled siRNA plus vehicle for T3. Age-matched mice without doxorubicin injury served as controls.

**Results:** In uninjured adult mice, transient therapy with DUSP5 siRNA and T3 increases cardiomyocyte numbers, which is required for the associated increase in LV contractile function, since both are blocked by danusertib. In mice with chronic doxorubicin injury, DUSP5 siRNA plus T3 therapy rebuilds LV muscle by increasing cardiomyocyte numbers, which reverses LV dysfunction and prevents progressive chamber dilatation.

**Conclusion:** RNA therapies are showing great potential. Importantly, a GMP compliant *in vivo*-jetPEI system for delivery of siRNA is already in use in humans, as is T3. Given these considerations, our findings provide a potentially highly translatable strategy for addressing doxorubicin cardiomyopathy, a currently untreatable condition.

## Introduction

Anthracyclines, including doxorubicin (Adriamycin), are part of many anticancer regimens because of their potent antitumor effect 1. They are used to treat a variety of malignancies, including more than 1 in 2 pediatric cancers 1. However, in young cancer survivors, the relative risk of congestive heart failure is 15 times higher than in their siblings who did not have cancer 2. The cumulative anthracycline dose is a major determinant of its cardiotoxicity, which results in the development of left ventricle (LV) dysfunction. Importantly, in pediatric cancer survivors, cardiovascular mortality surpasses cancer mortality 3. Extant clinical trials have mainly focused on its prevention 3, since once established, doxorubicin-induced cardiomyopathy is presently untreatable.

The mechanism of doxorubicin-induced cardiotoxicity involves topoisomerase-2β inhibition, which in cardiomyocytes causes double-stranded DNA breaks and, ultimately, many of these cells undergo p53-mediated apoptotic cell death 4. In patients exposed repeatedly to doxorubicin, elevated serum troponin levels indicate continuing cardiomyocyte death, whereas low serum troponin levels are associated with better outcomes 5. After repeated anthracycline exposure, gradual sub-clinical LV disease is observed in many pediatric cancer survivors, which manifests as dilated cardiomyopathy, LV posterior wall (PW) thinning and reduced fractional shortening 3. Therapy for this cardiomyopathy is non-specific and merely directed at heart failure management (e.g., diuretics and angiotensin converting enzyme inhibitors or angiotensin receptor blockers), since currently no treatments are available to enhance LV contractile function.

Current experimental approaches at cardiac regeneration, mostly genetic, are directed at increasing cardiomyocyte proliferation and improving LV ejection fraction when implemented acutely, either at the time of, or immediately after, an acute myocardial infarct 6-9. However, in the vast majority of patients who are diagnosed with heart failure it has already been present for some time. To our knowledge, efforts to improve cardiac contractile function by enhancing cardiomyogenesis to build heart muscle have not been attempted in the setting of severe chronic heart failure.

An important first step in developing a novel therapy is showing that it is effective in a preclinical model that faithfully recapitulates the human disease, which in this case involves demonstrating improvement in LV dysfunction in a mouse model of doxorubicin cardiomyopathy. Here we describe a pharmacological strategy, involving the use of thyroid hormone (T3) and DUSP5 siRNA, for stably building heart muscle by increasing the number of cardiomyocytes in uninjured adult mouse hearts, and then test its ability to treat LV dysfunction in an experimental model of chronic doxorubicin-induced cardiomyopathy.

We have previously shown that parenteral T3 administration increases cardiomyocyte numbers in neonatal murine hearts 10. The mechanism involves signaling by mitochondria-generated H_2_O_2_ acting via the redox sensor, peroxiredoxin-1, that activates c-Jun N-terminal kinase-2α2 (JNK2α2), a rare JNK isoform. JNK2α2 activation phosphorylates c-Jun, a component of the activator protein 1 (AP-1) early response transcription factor, resulting in enhanced IGF-1 expression and activation of proliferative ERK1/2 signaling. In young adult hearts (about 5 weeks old), however, T3 increases IGF-1 expression and IGF-1 receptor phosphorylation, but proliferative ERK1/2 signaling in cardiomyocytes is diminished and cardiomyocyte proliferation does not occur 11. The mechanism for this diminished ERK1/2 response involves the developmentally delayed expression in ventricular cardiomyocytes of the nuclear ERK1/2-specific dual specificity phosphatase, DUSP5, which starts at postnatal day-7 (P7) 11.

Here, we show that combining DUSP5 suppression with T3 therapy for a brief period of time produces a robust increase in p-ERK1/2 levels that induces cardiomyocyte proliferation in uninjured mouse hearts. Moreover, this therapeutic strategy is effective in repairing the hearts of young adult mice with doxorubicin-induced LV dysfunction.

## Methods

### Animal husbandry and mouse models

Mice were housed in pathogen-free conditions in an American Association for the Accreditation of Laboratory Animal Care-approved facility managed by the Department of Animal Resources of Emory University. All animal studies were approved by the Institutional Animal Care and Use Committee (IACUC) of Emory University, and performed in accordance with IACUC guidelines and regulations. We confirm that all experiments were performed in accordance with IACUC guidelines and regulations. C57BL/6 wild type (Jackson Laboratory, 000664) male and female mice were used for breeding and for the studies reported here. Animal husbandry was performed as described 10-11. For cardiomyocyte lineage tracing studies, we used Rosa26fs-Confetti[B6.129P2-Gt(ROSA)26Sortm1(CAG-Brainbow2.1)Cle/J, Jackson Laboratory, 017492] and Myh6-MerCreMer [B6.FVB(129)-A1cfTg(Myh6-cre/Esr1*)1Jmk/J, Jackson Laboratory, 005657] mice. We then generated double-transgenic Myh6-MerCreMer::Rosa26fs-Confetti mice by cross breeding. In the double transgenic mice, Cre recombinase causes the Brainbow 2.1 construct to recombine, which randomly labels cardiomyocytes either with GFP, CFP, RFP or YFP. We limited the extent of Cre-mediated recombination by adjusting the dose of 4-hydroxytamoxifen (H7904-5 mg, Sigma) to minimize replication-independent occurrences of adjacent cardiomyocytes of the same color.

Drugs such as T3, danusertib and 4-hydroxytamoxifen were administered, intraperitoneally (i.p.), to each mouse at the dose indicated. Vehicle was administered by the same route and animals thus treated served as controls. Phosphate buffered saline (PBS) was the vehicle for T3, 50%DMSO in PBS for danusertib, and soybean oil for 4-hydroxytamoxifen. DUSP5-specific siRNA or scrambled siRNA (control) was administered using *in vivo*-jetPEI (VWR, 89129-960). DUSP5 siRNA (100 µg) was dissolved in 1 ml of the *in vivo*-jetPEI:10% glucose mixture and was injected 100 µl per mouse i.p. (10 µg/mouse). DUSP5 siRNA (sc-60555) was a pool of 2 different siRNA duplexes (sc-60555A, sense: CAUGGCUUACCUCAUGAAtt and antisense: UUCAUGAGGUAAGCCAUGCtt; sc-60555B, sense: GACAGCUCCUUCAGUAUGAtt and antisense: UCAUACUGAAGGAGCUGUCtt; Santa Cruz Biotechnology). All sequences are provided in 5′→3′ orientation. We did not use any litters if one or more mouse in the litter was observed to be sick. Mice were first anesthetized with 5% isoflurane and then hearts harvested for further processing. Hearts were also collected at 40 h after T3 or vehicle treatment and ventricular cardiomyocytes prepared for immunoblotting or immunocytochemistry. In addition, hearts were collected post-therapy for immunocytochemistry, immunohistochemistry and cardiomyocyte number determination.

### Echocardiography analysis

Transthoracic echocardiography was performed as described 11. Briefly, echocardiography was performed under light isoflurane anesthesia on either DUSP5 siRNA or scrambled siRNA ± T3 treated mice using the Vevo 3100 imaging platform (VisualSonics Inc.) with a MX550D linear array transducer (axial resolution: 40 µm) and images were analyzed using Vevo Lab Desktop Software. We adjusted isoflurane anesthesia until heart rates were between 500-600 beats per minute. Both B-mode and M-mode images were collected to assess cardiac morphology and function. Parasternal short axis M-mode echocardiographic imaging was used to determine wall thicknesses and internal diameter of the LV at diastole and systole; fractional shortening was derived from these parameters. The intraventricular septum (IVS) function of M-mode (short axis) of Vevo Lab Desktop Software was used to determine these parameters. Endocardium and epicardium were outlined using the IVS function by tracing the anterior epicardial, endocardial and then posterior endocardial and epicardial wall. These measurements were made at the mid-apical level (half-way between the apex and the LV mid papillary level; ~1 mm from the apex) or mid-papillary level (~3.5 mm from the apex). Additionally, B-mode parasternal long axis images were acquired to determine LV long axis internal diameters and wall thicknesses. Using B-mode (long axis), the length of the LV chamber was determined by measuring the distance from the aortic root to the apical endocardial wall at end-systole and end-diastole. We also measured LVPW thickness at the apex using B-mode (long-axis) by tracing the apical wall from the epicardium to endocardium. LV volumes at end-diastole and end systole-measurements were calculated using the LV-trace function of the Vevo Lab Desktop Software in B-mode. LV endocardial borders were traced at end-diastole and end-systole by tracing the aortic root, apex, anterior wall, posterior wall and retracing the anterior and posterior wall until the myocardial wall is outlined. The Vevo Lab Desktop Software automatically determines volumes at end-diastole, end-systole, and calculates LV volume, stroke volume (SV) and LV ejection fraction (EF). Mice were randomized to different drug treatment groups using a preselect criteria of 28-38% EF. Mice with EF over or under this range were not included for drug randomization. At least three different images were taken for each cardiac parameter and measurements from these three individual images were averaged to acquire final measurement for that cardiac parameter.

### Cardiomyocyte number determination

The protocol for cardiomyocyte number determination was as described previously 10-11. Briefly, heparin (100-200 µl, 1000 USP units/ml) was injected intraperitoneally eight minutes prior to harvesting. Hearts were harvested under deep anesthesia (5% isoflurane). Hearts with their atria and aorta attached were washed with PBS and then the aorta cannulated for retrograde perfusion through the coronary circulation. Hearts were immediately perfused with cytofix (BD Biosciences, 554655) for 1 min. Subsequently, hearts were perfused with perfusion buffer (120 mM NaCl, 15 mM KCl, 0.5 mM KH_2_PO_4_, 5 mM NaHCO_3_, 10 mM HEPES, and 5 mM glucose, at pH 7.0) for 2 min and then with perfusion buffer containing collagenase type 2 (Worthington, LS004176) for 10-15 min at 37 °C. Perfusion and digestion buffers were freshly prepared, warmed to 37 °C and aerated with 5% CO_2_. Collagenase concentration was 2 mg/ml. After ~12 min of digestion, the atria were excised and the cardiac ventricles placed in a 6 cm dish containing 2 ml of digestion buffer; we then added ~2 ml of STOP buffer (perfusion buffer plus 10% bovine calf serum and 12.5 mM CaCl_2_). The ventricles were teased apart into small pieces followed by trituration through pipettes of progressively smaller diameters. The digested cardiomyocytes from each heart were collected in a 15 ml falcon tube and more STOP buffer was added to a volume of 10 ml. The final cell suspension was used to count cardiomyocytes using a hemocytometer. To avoid losses, cardiomyocytes were not purified, but could be readily identified by phase contrast microscopy based on their cytoplasmic size and rod shape. Four aliquots were counted per heart and the mean value was used to determine the total number of ventricular cardiomyocytes per heart.

For accurate cardiomyocyte number determination, a critical step is optimal digestion efficiency and operator-specific variability in isolation and counting. We have eliminated operator-specific variability by using the same operator between experiments. Heart digestion is chiefly dependent on collagenase concentration in the perfusion medium, its activity, exposure time and temperature. To optimize digestion efficiencies, these variables were adjusted for each group of mice depending on the age of the mouse. Importantly, collagenase activity was kept uniform between the biological replicates and across experiments by using the same lot of enzyme. Additionally, a brief fixation with cytofix (~1 min) before starting perfusion with collagenase was used to maintain cardiomyocyte structure and to prevent the generation of fragmented cardiomyocytes. Cardiomyocytes during the postnatal stages analyzed are much larger than non-myocytes and are readily identifiable due to their cytoplasmic size and rod-shape 10. Digestion efficiency was calculated [ventricular weight %, determined by (original weight - residual)/original weight] after each change in condition. We found that maximal digestion efficiencies were between ~97% and 99%. Upon microscopic examination, the residual tissue was almost entirely undigested cardiac valves and blood vessels. Over-digestion neither improved digestion efficiencies, nor did it increase cardiomyocyte yield. We did not estimate cardiomyocyte numbers from under-digested hearts in which disaggregation of myocardial tissue was incomplete. Suboptimal cannulation of the aorta was the cause of under-digestion but was infrequent.

### Cardiomyocyte isolation for immunocytochemistry and immunoblotting

For immunoblotting and immunocytochemistry, hearts were enzymatically digested as described above. Before making single cell suspensions, atria were excised, and the LVs were trimmed from the RV. Cardiomyocytes were purified with three low speed centrifugations (18 x g for 4 min at room temperature), which caused cardiomyocytes to settle as a pellet. Supernatant fractions, enriched in non-myocytes, were discarded. Resulting cardiomyocyte preparations were > 95% pure. Aliquots of cardiomyocytes were snap frozen in liquid nitrogen and stored at -80 °C for immunoblotting. Additionally, aliquots were fixed with Cytofix (BD Biosciences, 554655) for 5 min and spread on glass slides for immunocytochemistry.

### Immunofluorescence

Immunofluorescence was performed as described earlier 10-11. Briefly, cardiomyocytes were isolated as described above and fixed in Cytofix (BD Biosciences, 554655) for 5 min. After pre-blocking, cardiomyocytes were stained with anti-cardiac troponin T- (Miltenyi Biotec, 130-119-674), in 10% v/v goat serum. DAPI was used to stain nuclei. High power (40X) images were acquired on a Leica Microsystems DMZ6000 microscope by using the LAX program and analyzed using Nikon's NIS Elements advanced fluorescence program, to determine cardiomyocyte size.

### Immunohistochemistry

Immunohistochemistry was performed as described previously 12. Briefly, mouse hearts were immersion-fixed in 10% formalin and then stored in 70% ethanol until paraffin embedding and sectioning. Paraffin-embedded sections (~7 μm) were mounted on +/+ glass slides and were deparaffinized with xylene and then rehydrated in ethanol. Antigen retrieval was performed using sodium citrate buffer. The mounted sections, immersed in sodium citrate buffer, were brought to 100 °C intermittently over 3 min using a microwave oven. This step is repeated three more times. Sections were blocked with 10% v/v goat serum for 30 min at room temperature before applying primary antibody: wheat germ agglutinin ((WGA)-Alexafluor 647 conjugate (W32466, ThermoFisher) to identify cell borders. After a 60 min incubation at room temperature sections were washed three times for five minutes each with PBS, and then coverslips were mounted using mounting media containing DAPI (4′,6-diamidino-2-phenylindole) to identify nuclei. High power (63X) images were acquired on a Leica Microsystems DMZ6000 microscope using the LAX program and analyzed using Nikon's NIS Elements advanced fluorescence program.

### Histology

Heart sections, prepared and processed as described above, were stained using a trichrome stain kit (ab150686, Abcam) as per manufacturer's protocol. High power images were acquired and processed using Hamamatsu's NanoZoomer-SQ Digital slide scanner and NDP.view2 Viewing software. Fibrosis was quantified using Nikon's Elements 3.0 Advanced Fluorescence program.

### Apoptosis Analysis

Heart sections prepared and processed as described above. Apoptosis assay was performed by using the Click-iT Plus TUNEL Assay kit for *In situ* Apoptosis Detection (Thermo Fisher Scientific) as per the manufacturers' protocol that is based on the principle of terminal deoxynucleotidyl transferase (TdT)-mediated dUTP nick end-labeling (TUNEL).

### Immunoblotting

The protocol used was as described previously 10-11. Whole cell cardiomyocyte lysates were generated by re-suspending cardiomyocytes in 250 µl of RIPA buffer (Cell Signaling, 9806S) supplemented with phosphatase inhibitor cocktail 2 and 3 (Sigma-Aldrich, P5726-1ML and P0044-1ML), 0.1 mM phenylmethylsulfonyl fluoride (PMSF, Sigma-Aldrich, 93482-50ML-F) and protease inhibitor cocktail (Roche, 11697498001). Cardiomyocytes were lysed by sonication and then pelleted by centrifugation at 21,000 x g for 30 min. The resulting supernatant fractions were aliquoted into fresh Eppendorf tubes and then snap-frozen in liquid nitrogen. Aliquots stored at -80 °C, were allowed to thaw on ice immediately before use. Initially, a 5 to 10 µl aliquot (~20 μg protein) was mixed with an equal volume of 2× Laemmli sample buffer (Bio-Rad, 1610737), heated for 5 min at 95-99 °C and then immediately cooled on ice for 5 min. The samples were then centrifuged briefly before loading onto and fractionation by SDS-polyacrylamide gel (12-18%) electrophoresis (SDS-PAGE), which was performed at 200 volts for 5 min, and then at 150 volts for 30 min to 2 h. Proteins thus resolved were then transferred to a PVDF membrane by electroblotting (Turbo Transfer; Bio-Rad). Depending upon the molecular weight of the proteins or protein complexes, the transfer time was varied for high (11 min), medium (7 min) and low (5 min) molecular weight proteins. After transfer, all blots were pre-blocked for 30-60 min in Superblock (Thermo Scientific, 37536). Initially, the samples were probed with GAPDH antibody. Based on GAPDH, loading of each sample was adjusted so that all samples contained an equal amount of GAPDH. Membranes were probed with the target protein-specific primary antibody. For quantitative analysis, the membrane was then stripped and re-probed with GAPDH to ensure that loading was normalize for each sample. For stripping, the membrane was washed twice with 1x Tris-buffered saline (TBS, Thermo Scientific, BP2471-1) for 5 min each and then incubated with Restore™ Western Blot Stripping Buffer (Thermo Scientific, 21059) for 5-15 min and then washed again twice with 1× TBS and pre-blocked with Superblock (37536, Thermo Scientific) for 1 h before incubating with GAPDH antibody. Primary antibodies (see below) were also diluted in Superblock and incubated for 2 h at 22 °C, or overnight at 4 °C, followed by horseradish peroxidase (HRP)-labeled secondary antibody (1:10,000) for 45 min at 22 °C. The signals were detected using Super Signal West Dura Detection Reagent (Thermo Scientific, 34075) and images captured on a Bio-Rad GelDoc system equipped with CCD camera and ImageLab program (Bio-Rad). Quantification was performed by densitometry using the ImageLab program. Antibodies used for immunoblots are DUSP5 (ab200708, Abcam), ERK1/2 (4695, Cell Signaling), p-ERK1/2 (4370, Cell Signaling), cyclin D1 (ab134175, Abcam) and GAPDH (2118, Cell Signaling). Most of these antibodies are profiled in 1DegreeBio and were additionally validated using siRNA knockdown.

### Primary cardiomyocyte culture

Cardiac ventricles from P1-P3 mice were enzymatically disaggregated using gentleMACs Dissociator (Miltenyi Biotec) as described 10. Cardiomyocytes were separated from non-myocytes as described 10. Cardiomyocytes and non-myocytes were separately cultured at 37 °C in a 5% CO_2_ humidified incubator (Hera cell 240, Thermo Scientific) on collagen pre-coated 12-well culture dishes initially in serum containing media for 12 hours and changed to serum-free media as described 10. Cardiomyocytes and non-myocytes were harvested 8 h after exposure to 10 nM T3 or vehicle and stored at -80 °C until use for immunoblotting as described 10.

### Statistical analysis

Statistical significance of data was determined using Graphpad Prism 8. The Shapiro-Wilk test was used to determine if the data were normally distributed; in this case, we used one-way ANOVA followed by Sidak multiple comparisons test, or unpaired two-tailed Student's t-test for comparisons involving 2 groups. For estimation of variance, the F-test was used when comparing 2 groups and the Brown-Forsythe test was used when comparing multiple groups by 1-way ANOVA. *P*-values < 0.05 were considered significant. Results are expressed as mean ± s.e.m. This study did not involve Bayesian analysis, information on the choice of priors and Markov chain Monte Carlo settings. This study also did not require hierarchical and complex designs, identification of the appropriate level for tests and full reporting of outcomes. Estimates of effect sizes were also not utilized in this study.

## Results

### DUSP5 siRNA+T3 stimulates *in vivo* cardiomyocyte proliferation

T3-stimutated cell proliferation, *in vitro*, in neonatal cardiomyocytes, is mediated by IGF-1 10. We show here that, *in vivo*, in mature cardiomyocytes (from 5-week-old mice), 3 doses of T3 (2 ng/g/day, i.p.) increased IGF-1 expression in LV cardiomyocytes, but not in non-myocytes (Figure S1A). This selective *in vivo* effect of T3 was also found *in vitro* in isolated cultured cardiomyocytes versus non-myocytes from neonatal hearts (Figure S1B). We have previously shown that, despite this increase in IGF-1 in mature cardiomyocytes, a concomitant increase in p-ERK1/2 levels is not observed as it is inhibited by a developmentally-regulated increase in the ERK1/2-specific phosphatase DUSP5 11. We, therefore, tested the hypothesis that combining DUSP5 suppression with T3 therapy will stimulate ERK1/2 phosphorylation and cell proliferation in mature LV cardiomyocytes. We used *in vivo*-jetPEI to deliver two daily doses of DUSP5 siRNA or DUSP5 scrambled siRNA (control) to ~5-week-old male mice. DUSP5 siRNA, but not scrambled siRNA, reduced DUSP5 levels in cardiomyocytes by ~98% (*P* < 0.001) (Figure S2A-C). T3 (4 ng/g, i.p.), when combined with DUSP5 siRNA therapy, increased cardiomyocyte p-ERK1/2 levels by ~2-3-fold relative to levels in mice pretreated with scrambled siRNA+T3 (*P* < 0.001) (Figure S2B-C).

T3, administered intraperitoneally in the mouse, has a half-life in the circulation of ~12 h 13. Because sustained ERK1/2 activation is critically important for cell proliferation 14, we administered DUSP5 siRNA daily over 2 days and then gave T3 daily (2 ng/g/day, i.p.) over 5 days. The dose and duration of T3 was determined empirically. We titrated T3 dosing until a dose that was just sufficient to acutely (within 2 days) increase cyclin B1 expression in cardiomyocytes, *in vivo*, was achieved. Cyclin B1, the regulatory subunit of cyclin-dependent kinase 1, is essential for mitosis entry and progression 15. This T3 dose, when given daily over 5 days, was sufficient to increase cardiomyocyte numbers, but administration for only 2 days was not. These empirical data formed the basis of the T3+DUSP5 siRNA dosage protocol described by us previously 11. This T3 dosing protocol increased circulating T3 levels twofold (serum T3 levels were 3.5 ± 0.19 ng/ml and 1.67 ± 0.02 ng/ml 12 h after the 5^th^ T3 and vehicle dose, respectively; *P* < 0.001). IGF-1 expression in cardiomyocytes increased 24 h after the start of T3 dosing and remained elevated at 24 h after the final T3 dose (Figure S3A-C). p-ERK1/2 and cyclin B1 levels increased similarly after the start of T3 dosing, their decay followed that of IGF-1 (Figure S3A-C).

**Figure 1:**
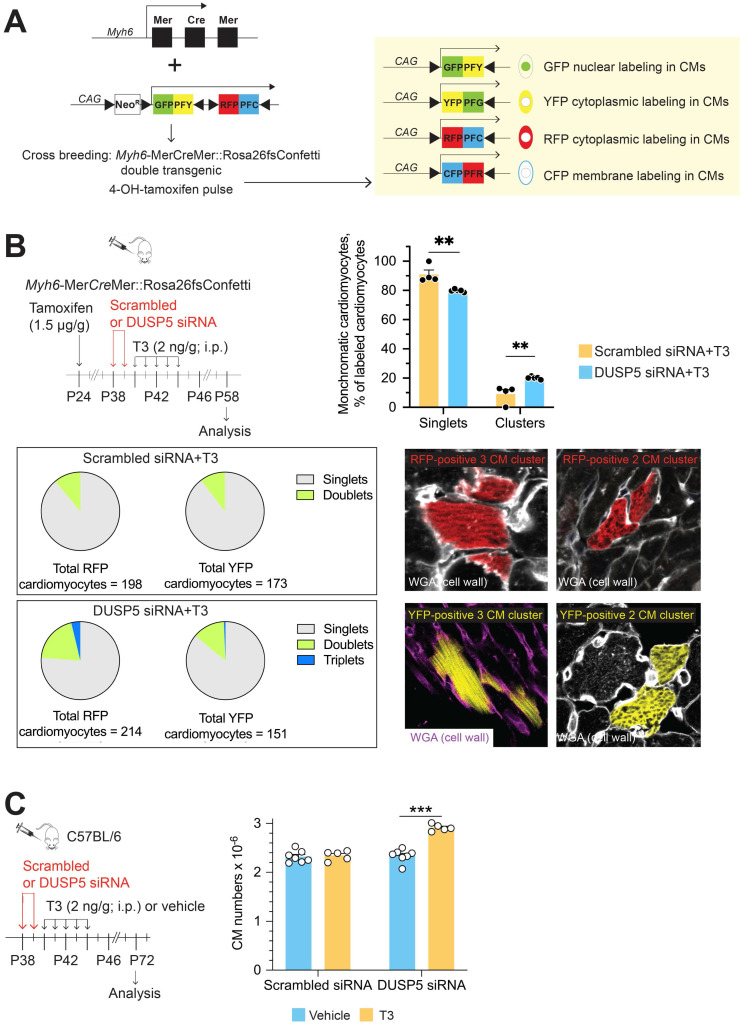
** Transient DUSP5 siRNA+T3 therapy stimulates cardiomyocyte proliferation and number expansion.** (A) Schematic shows the Confetti construct (left) and potential for expression of green (GFP), yellow (YFP), red (RFP) and cyan fluorescent protein (CFP) in cardiomyocytes (right) following Cre-mediated recombination. (B), Schematic (left) shows the experimental protocol for 4-hydroxytamoxifen treatment to initiate Cre-mediated recombination for expression of fluorescent proteins in cardiomyocytes and timing of DUSP5 siRNA and T3 therapy. Bar graph (right) shows quantification of monochromatic singlet or clustered cardiomyocytes in DUSP5 scrambled siRNA+T3 and DUSP5 siRNA+T3 treated mice (*n* = 4-5 mice per group); A pie chart analysis (below left) of either red (RFP) or yellow (YFP) colored monochromatic singlets, doublets and triplets distribution in DUSP5 siRNA+T3 or scrambled siRNA+T3 treated mice shows that Triplets (3-cell clusters) only occur in DUSP5 siRNA+T3 treated mouse hearts. Representative images (below right) of monochromatic cardiomyocyte (CM) clusters with cell outlines delineated using wheat germ agglutinin (WGA) staining. (C) Schematic (left) shows the experimental protocol. Bar graph (right) shows ventricular cardiomyocyte numbers analysis after *in vivo* DUSP5 scrambled siRNA, DUSP5 scrambled siRNA+T3, DUSP5 siRNA or DUSP5 siRNA+T3 therapy (*n* = 5-7 mice per group). **P < 0.01, ***P < 0.001. Individual data points and mean ± s.e.m are shown. Comparisons were made using a 2-tailed *t*-test (*B*) or ANOVA with Sidak multiple comparison test (*C*).

We then used multicolor clonal analysis in double-transgenic *Myh6*-Mer*Cre*Mer::Rosa26fs-Confetti mice 16 to evaluate replication as well as the proliferative strategies of individual LV cardiomyocytes after DUSP5 siRNA+T3 combination therapy. A schematic of the Confetti construct is shown in Figure 1A. In the double-transgenic *Myh6*-Mer*Cre*Mer::Rosa26fs-Confetti mice, Cre recombinase activation upon 4-hydroxytamoxifen administration causes the Brainbow 2.1 construct to recombine, which randomly labels cardiomyocytes either with GFP, CFP, RFP or YFP. If 4-hydroxytamoxifen treatment, followed several days later by the administration of a mitogenic stimulus, results in formation of large multicell monochromatic cardiomyocyte clusters (adjacent cells displaying the same fluorescent marker), it would signify cardiomyocyte replication due to clonal expansion of cardiomyocytes. By contrast, when single replication events occur, the outcome is the generation of a 2-cell monochromatic cardiomyocyte cluster. Quantifying the frequency of replication-driven 2-cell monochromatic clusters and, visually identical, 2-cell monochromatic clusters that can arise because of chance homologous recombination events (which are possible after the initial administration of 4-hydroxytamoxifen) is necessary to document *in vivo* cardiomyocyte replication. In this assessment, replication-driven monochromatic clusters represent the signal and chance homologous recombination-driven clusters represent the noise. Optimization of this lineage tracing technique requires increasing the signal-to-noise ratio.

Theoretically, the frequency of homologous recombination-driven monochromatic cardiomyocyte clusters is proportional to the frequency of fluorescent labeling. We titrated the dose of 4-hydroxytamoxifen to achieve minimal labeling of cardiomyocytes. These 4-hydroxytamoxifen titration studies were performed in mice that were subsequently given scrambled siRNA+T3, which serves as a control to the mitogen challenge that uses DUSP5 siRNA+T3 therapy. As shown in Figure S4A-H, the frequency of RFP, YFP, CFP and GFP labeled cardiomyocytes increased markedly from undetectable levels at 1.0 µg 4-hydroxytamoxifen/g body weight to ~67% at 20 µg/g. At 1.5 µg 4-hydroxytamoxifen/g, the frequency of individual monochromatic cardiomyocytes was < 1%, which we considered to represent a minimal level of labeling.

We also assessed the impact of 4-hydroxytamoxifen dose on the expression of RFP, YFP, GFP and CFP. Many mature murine cardiomyocytes are binucleated. Thus, if distinct recombination events occur in each of the 2 nuclei some cardiomyocytes will become bichromatic (labeled with two colors), which has the potential to complicate lineage-tracing analyses. At higher doses of 4-hydroxytamoxifen several cardiomyocytes became bichromatic but, at a dose of 1.5 µg/g, bichromatic cardiomyocytes were not seen (Figure S4F). While all four fluorescent tags were observed at higher doses of 4-hydroxytamoxifen, at 1.5 µg/g we could only detect YFP- and RFP-labelled cardiomyocytes; the frequency of these individual labeling events was 0.92% (Figure S4G) and 0.93% (Figure S4H) of all cardiomyocytes, respectively. Based on these optimization studies, we used 1.5 µg 4-hydroxytamoxifen/g body weight for subsequent lineage-tracing studies.

To address if DUSP5 siRNA+T3 therapy increases cardiomyocyte replication *in vivo* we first administered 4-hydroxytamoxifen. Two weeks later, we administered DUSP5 or scrambled siRNA to* Myh6*-Mer*Cre*Mer::Rosa26fs-Confetti mice over 2 days, followed by 5 daily doses of T3 (2 ng/g, i.p.). At the time of siRNA treatment, the mice were 5-weeks-old. Hearts were harvested 2 weeks later; multiple LV tissue sections from each heart, representing the entire LV, were studied. In control mice, treated with scrambled siRNA+T3, monochromatic clustered and singlet cardiomyocytes were 9% ± 3.04% and 91% ± 3.04%, respectively. By contrast, in DUSP5 siRNA+T3 treated mice, monochromatic clustered and singlet cardiomyocytes were 20.1% ± 0.42% and 79.9% ± 0.42%, respectively, indicating a 2.2-fold difference (*P* = 0.005) in the relative frequency of monochromatic clustered cardiomyocytes (Figure 1B). In these treatment groups, monochromatic clusters were found throughout the LV. In Figure 1B, we also present data as pie charts, separately for each color showing the relative differences in singlet cardiomyocytes as well as 2- and 3-cell monochromatic clusters expressing either RFP or YFP. These data reveal that 3-cell monochromatic clusters were only found in DUSP5 siRNA+T3-treated *Myh6*-Mer*Cre*Mer::Rosa26fs-Confetti mice (Figure 1B). The total increase in monochromatic cluster frequency, as well as occurrence of larger (3-cell) monochromatic clusters, suggests that DUSP5 siRNA+T3 therapy increases *in vivo* cardiomyocyte replication events. The absence of very large monochromatic cardiomyocyte clusters argues against the scenario in which DUSP5 siRNA+T3 therapy stimulates clonal expansion of a few highly proliferative cardiomyocytes, as seen in the zebrafish heart 17. Collectively, our data supports the notion that DUSP5 siRNA+T3 therapy stimulates limited proliferation in multiple cardiomyocytes throughout the LV.

The lineage-tracing technique likely underestimates the extent of DUSP5 siRNA+T3-driven cardiomyocyte proliferation because random adjacent recombination events clearly contribute to a subset of 2-cell clusters, increasing the baseline number of 2-cell clusters. We, therefore, sought a direct method to determine the extent of cardiomyocyte proliferation that results from DUSP5 siRNA+T3 therapy. For these studies, we gave 2 daily doses of DUSP5 siRNA followed by 5 daily doses of T3 (2 ng/g, i.p.). Four weeks after therapy, hearts were enzymatically disaggregated and cell suspensions created from the cardiac ventricles 10,11. Cardiomyocytes in these cell suspensions, identified by their size and by rod shape, were counted using a hemocytometer. We found that there were 25% more ventricular cardiomyocytes in DUSP5 siRNA+T3-treated mice than in mice treated with DUSP5 siRNA alone (*P* < 0.001), or after scrambled siRNA, with or without T3 therapy (*P* < 0.001, in each case) (Figure 1C). We also determined cardiomyocyte sizes and found that the DUSP5 siRNA+T3-induced increases in cardiomyocyte numbers was associated with a reduction in the average cardiomyocyte cell size (by 12% for mononuclear cells, *P* < 0.05, and by 15% for binuclear cells, *P* < 0.001) (Figure S5). Collectively, these data show that DUSP5 siRNA+T3 therapy increases ventricular cardiomyocyte numbers without inducing cardiomyocyte hypertrophy.

### Cardiomyocyte proliferation underlies increased LV growth and contractile function

DUSP5 siRNA+T3 therapy increased ventricle-to-body weight and LV-to-body weight ratios by 15% and 25%, respectively, at 2-weeks post-therapy (*P* < 0.001), without influencing the growth of other heart chambers (e.g., right ventricle and atria), of other organs (e.g., the liver, lungs, and the kidneys) or body weights (Table 1). By contrast, neither T3 nor DUSP5 siRNA monotherapy altered LV-to-body weight ratios. Thus, administration of DUSP5 siRNA plus T3 selectively increases LV growth.

**Figure 2:**
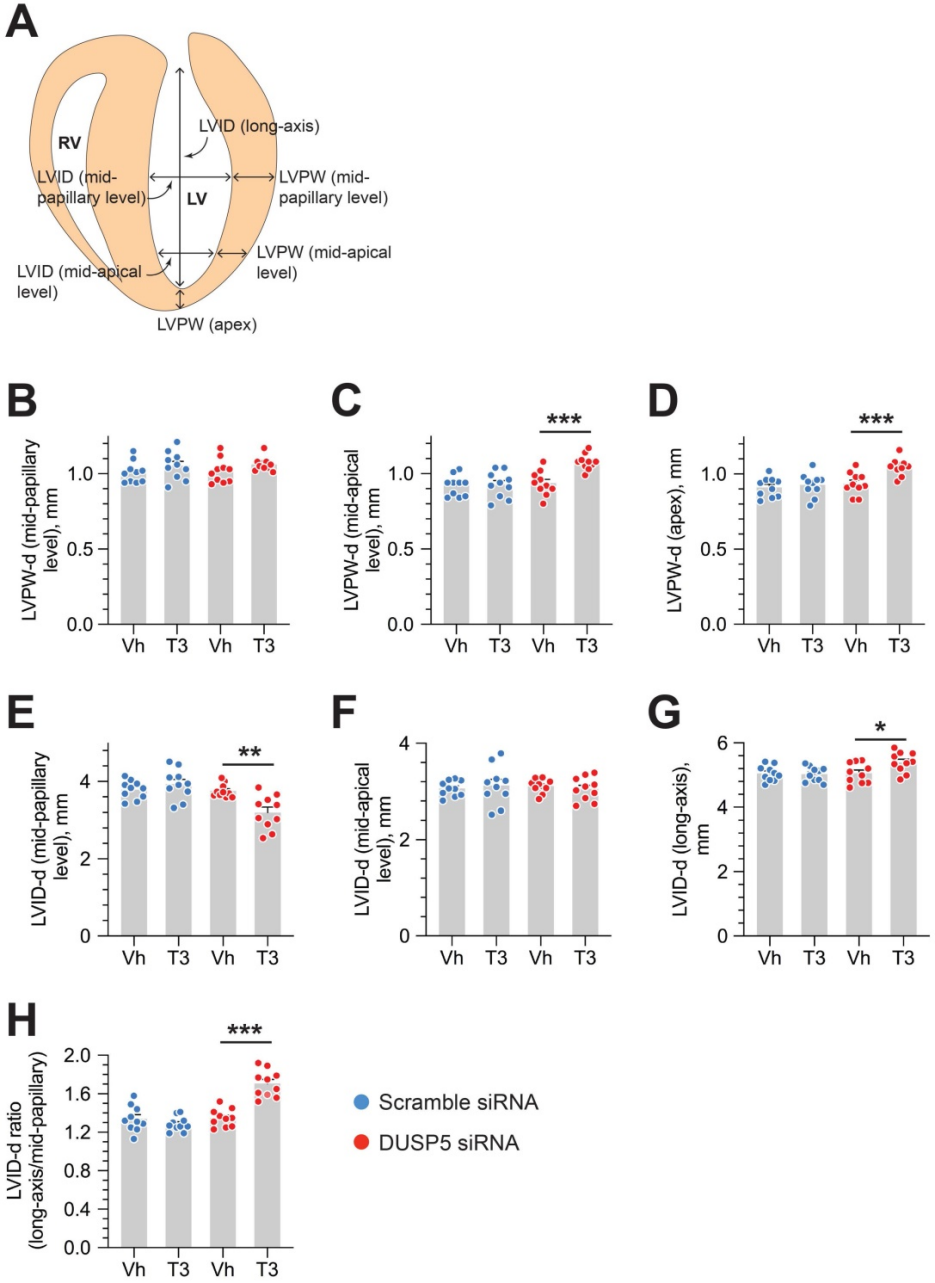
** DUSP5 siRNA+T3 therapy induces LV growth and LV chamber elongation in young adult (5-week-old) mice.** (A) Diagram showing sites of LV dimension measurements using echocardiography. (B-G) Effect of T3 therapy in mice pretreated with either DUSP5 siRNA or DUSP 5 scrambled siRNA (control) on posterior wall (PW) dimensions at diastole at the LV mid-papillary (B) and mid-apical level (C) and at the LV apex (D), and LV chamber internal dimensions (LVID) at diastole at the LV mid-papillary (E) and mid-apical level (F) and LVID long axis (G). (H) LVID long-axis-to-mid-papillary ratio at diastole (*n* = 10 mice per group); *P < 0.05, **P < 0.01, ***P < 0.001. Individual data points are mean ± s.e.m are shown. Comparisons were made using ANOVA with Sidak multiple comparison test; only within group *P*-values are shown.

**Figure 3:**
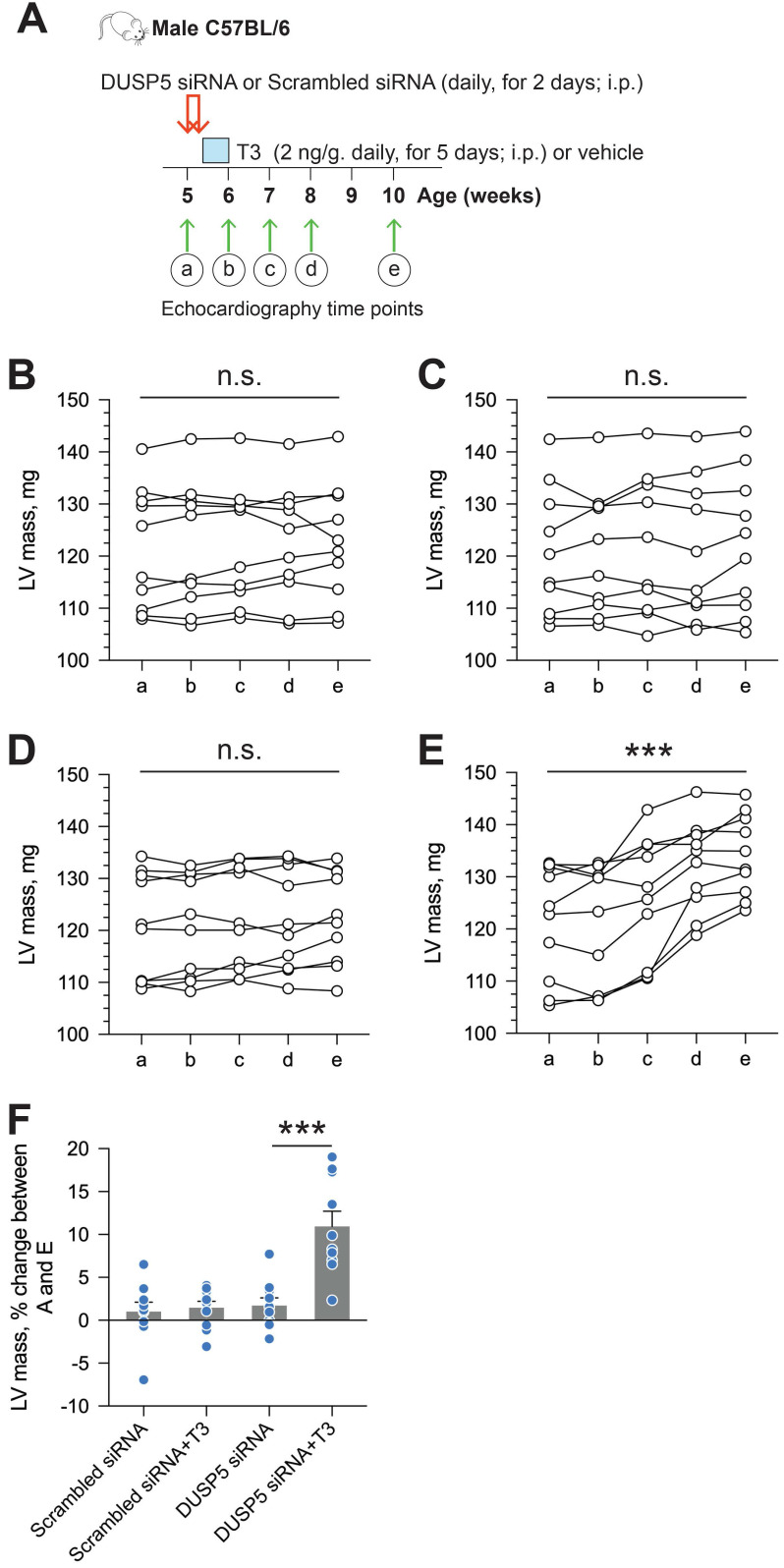
** LV mass increases progressively after DUSP5 siRNA+T3 therapy in young adult (5-week-old) mice.** (A) Diagram showing the time points before (a) and after DUSP5 siRNA+T3 therapy (b to e) at which LV mass was determined using echocardiography. (B-E) Serial determinations of LV mass before and after therapy with scrambled siRNA (control) (B), DUSP5 scrambled siRNA+T3 (C), DUSP5 siRNA (D) and DUSP5 siRNA+T3 (E) (*n* = 10 mice per group). Comparisons were made using a paired 2-tailed *t*-test between time points A and E. ***P < 0.001. (F) Relative change (%) in LV mass between the start of therapy and 4-week after therapy (*n* = 10 pairs per group). Comparisons were made using ANOVA with Sidak multiple comparison test; only within group *P*-values are shown. Individual data points and mean ± s.e.m are shown. ***P < 0.001.

We used parasternal short-axis M-mode and long-axis B-mode echocardiographic imaging to determine LV dimensions and LV contractile function. Multiple imaging modalities allowed us to assess LV remodeling by determining PW dimensions at the LV mid-papillary and mid-apical level as well as at the LV apex (Figure 2A). These parameters were determined four weeks after administering 2 daily doses of DUSP5 siRNA followed by 5 daily doses of T3 (2 ng/g, i.p.). Relative to DUSP5 siRNA monotherapy, DUSP5 siRNA+T3 therapy resulted in an increase in PW thickness at the LV apex and the mid-apical level, but not at the mid-papillary level (Figure 2B-D). DUSP5 siRNA+T3-driven remodeling also resulted in a longer and narrower LV cavity, based on measurements of minor and major axis LV internal dimensions at diastole (LVID-d) (Figure 2E-H). Consistent with these changes, LV mass increased by ~10% (*P* < 0.001) (Figure 3A-F). Of note, however, the formula used by the Vevo software employed here to calculate LV mass using M-mode imaging [1.05 × ((LVID-d + LVPW-d + IVS-d)^3^ - LVID-d^3^) mg] assumes constant LV wall thickness (based on measurements at the mid-papillary level). Because of uneven changes in LV PW after DUSP5 siRNA+T3 therapy the LV mass measurement is an underestimate; thus, we found that DUSP5 siRNA+T3 therapy increased LV mass by ~10%, but LV-to-body weight ratio increased by ~25%. In contrast to the effects of DUSP5 siRNA+T3 therapy, T3 had no discernable effects when combined with scrambled siRNA therapy (Figure 3A-F). Sequential temporal analysis showed that LV mass gradually increases after cessation of DUSP5 siRNA+T3 therapy, with major changes occurring between 1 and 2 weeks after therapy (Figure 3A-F).

**Figure 4:**
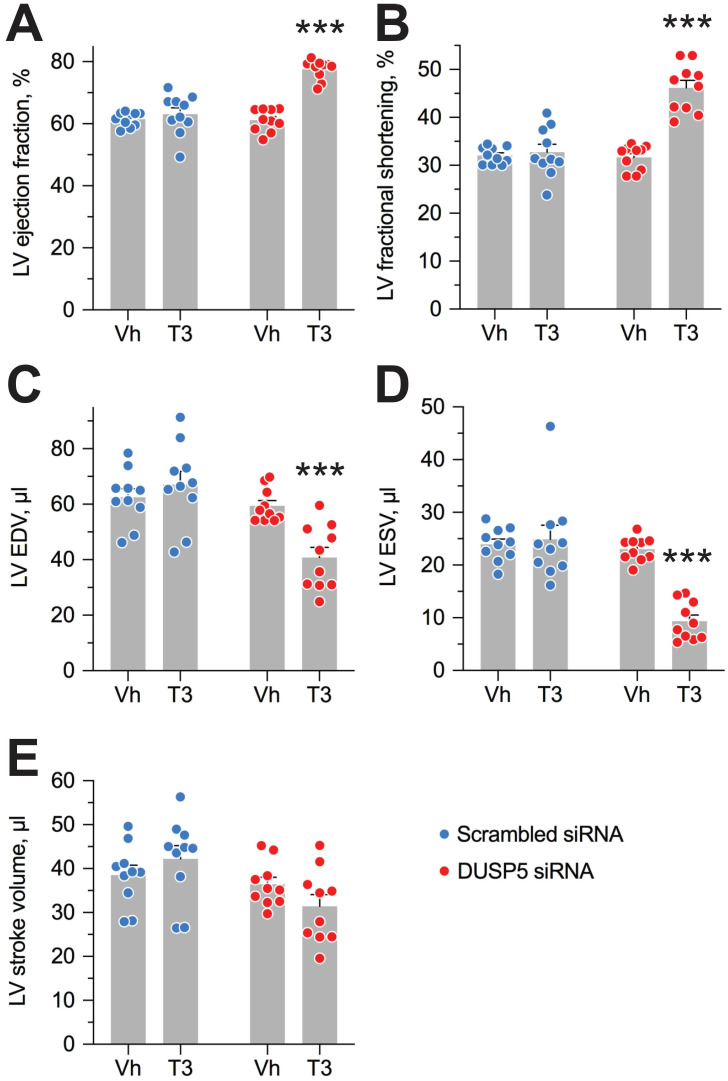
** DUSP5 siRNA+T3 therapy potentiates LV contractile function in young adult (5-week-old) mice.** (A-E) Effect of T3 therapy in mice pretreated with either DUSP5 siRNA or DUSP5 scrambled siRNA (control) on LV ejection fraction (A), LV fractional shortening (B), LV end-diastolic volume (EDV) (C), LV end-systolic volume (ESV) (D) and LV stroke volume (E) (*n* = 10 mice per group). Individual data point and mean ± s.e.m are shown. Comparisons were made using ANOVA with Sidak multiple comparison test; only within group *P*-values are shown. ***P < 0.001.

Evaluation of LV volumes at end-diastole (EDV) and end-systole (ESV), four weeks after DUSP5 siRNA+T3 therapy showed an ~14-percentage point increase in ejection fraction [(EDV - ESV) x 100/EDV] (*P* < 0.0001) (Figure 4A). In keeping with this increase, LV fractional shortening increased ~45% (*P* < 0.0001) (Figure 4B). DUSP5 siRNA+T3 therapy decreased EDV by ~30% (*P* < 0.0001) (Figure 4C). Because of an even greater decrease in ESV (by 60%; *P* < 0.0001) (Figure 4D), stroke volume (EDV - ESV) was not significantly changed (Figure 4E). By contrast, scrambled siRNA therapy, with or without T3, was without effect (Figure 4A-E). Representative videos showing LV trace of parasternal long-axis B-mode images of mouse hearts between full diastole and full systole after therapy with scrambled siRNA+T3 (Video S1) or DUSP5 siRNA+T3 (Video S2) are provided. Thus, collectively, our data show that DUSP5 siRNA+T3 therapy induces cardiomyocyte number expansion, which results in a LV PW that is more muscular and has enhanced contractility.

**Figure 5:**
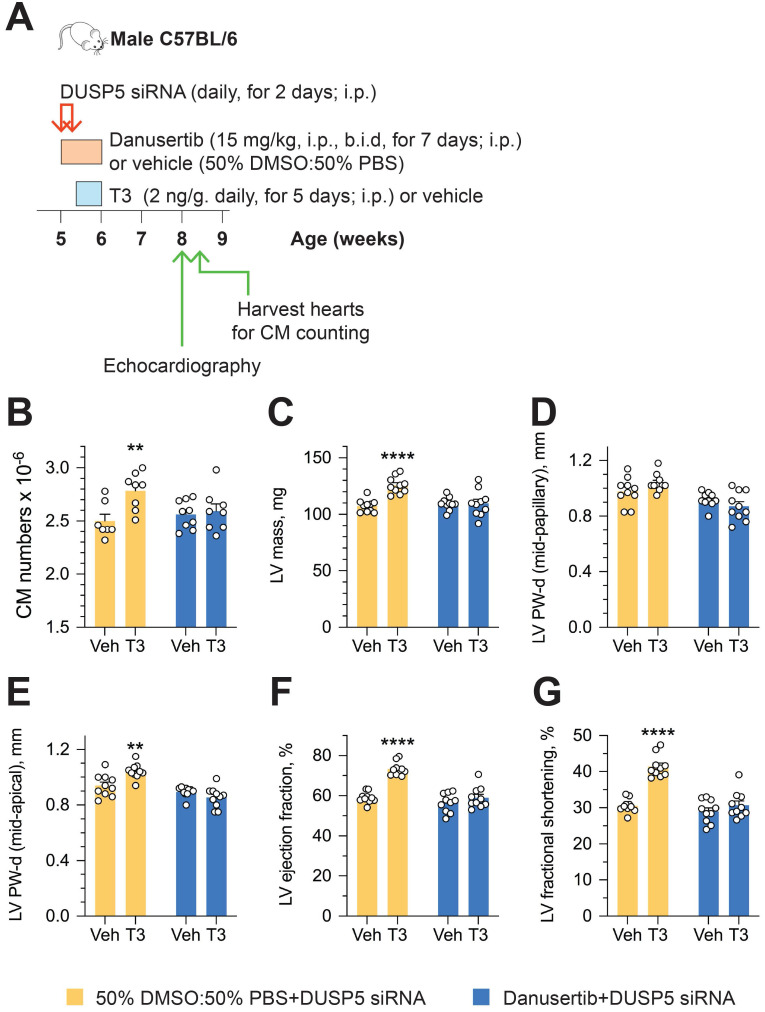
** Cardiomyocyte number expansion drives LV growth and increases in LV contractile function after DUSP5 siRNA+T3 therapy.** (A) Experimental protocol for testing the effects of the cell cycle inhibitor, danusertib, on T3-stimulated increases in LV dimensions and contractile function in DUSP5 siRNA-treated mice. (B) T3-stimulated increase in ventricular cardiomyocyte numbers in DUSP5 siRNA+T3 treated mice are blocked by danusertib (*n* = 6-8 mice per group); **P < 0.01. (C-G) *In vivo* echocardiographic evaluation of T3-stimulated changes in LV mass (C), LV PW dimensions, at diastole, at the LV mid-papillary level (D) and at the LV apex (E), LV ejection fraction (F) and LV fractional shortening (G) in DUSP5 siRNA+T3 treated mice, with or without danusertib treatment (*n* = 10 mice per group); **P < 0.01, ****P < 0.0001. Individual data points and mean ± s.e.m are shown. Comparisons were made using ANOVA with Sidak multiple comparison test; only within group *P*-values are shown (B-G).

To determine if an increase in cardiomyocyte number is central to sustained changes in LV dimensions and contractile function, we studied the effect of danusertib - a potent, small molecule inhibitor of aurora kinases that blocks the cell cycle by interfering with the normal progression of mitosis 18 - on DUSP5 siRNA+T3-driven changes in LV remodeling and contractile function. Danusertib (15 mg/kg, i.p., b.i.d) or its vehicle (50% DMSO:50% PBS) were administered daily for seven days, beginning at the start of DUSP5 siRNA treatment. Immediately after the 2-days of DUSP5 siRNA treatment, mice received T3 or vehicle therapy for 5 days, as above. LV dimensions and function were assessed 2 weeks after the end of therapy and cardiomyocyte numbers were determined 24-48 hours later (Figure 5A). Danusertib not only inhibited DUSP5 siRNA+T3-induced increases in cardiomyocyte numbers (Figure 5B), LV mass (Figure 5C) and LV PW thickness (Figure 5D-E), but also increases in LV ejection fraction (Figure 5F) and fractional shortening (Figure 5G). These data imply that DUSP5 siRNA+T3-driven expansion of the LV myocardium and improvements in contractile function require cardiomyocyte proliferation.

### DUSP5 siRNA+T3 rebuilds the LV myocardium in chronic doxorubicin-injured hearts

In preliminary experiments, we found that TUNEL staining in LV cardiomyocytes was increased by ~150-fold (*P* = 0.02) within 48 hours after doxorubicin (20 µg/g, i.p.) administration, indicating marked cardiomyocyte apoptosis (Figure S6A-B). Given that girls are at greater risk of developing LV dysfunction after anthracycline therapy than boys 19, we studied the effects of repeated doxorubicin exposure on the LV of young female mice. Five-week-old female mice were given 3 doses of doxorubicin (20 µg/g, i.p.) at 2-weekly intervals, starting at 5-weeks-of-age. The effect of DUSP5 siRNA+T3 therapy as well as control, scrambled siRNA therapy, was studied over 3 weeks. A group in which mice were neither exposed to doxorubicin nor therapy served as age-matched controls.

**Figure 6:**
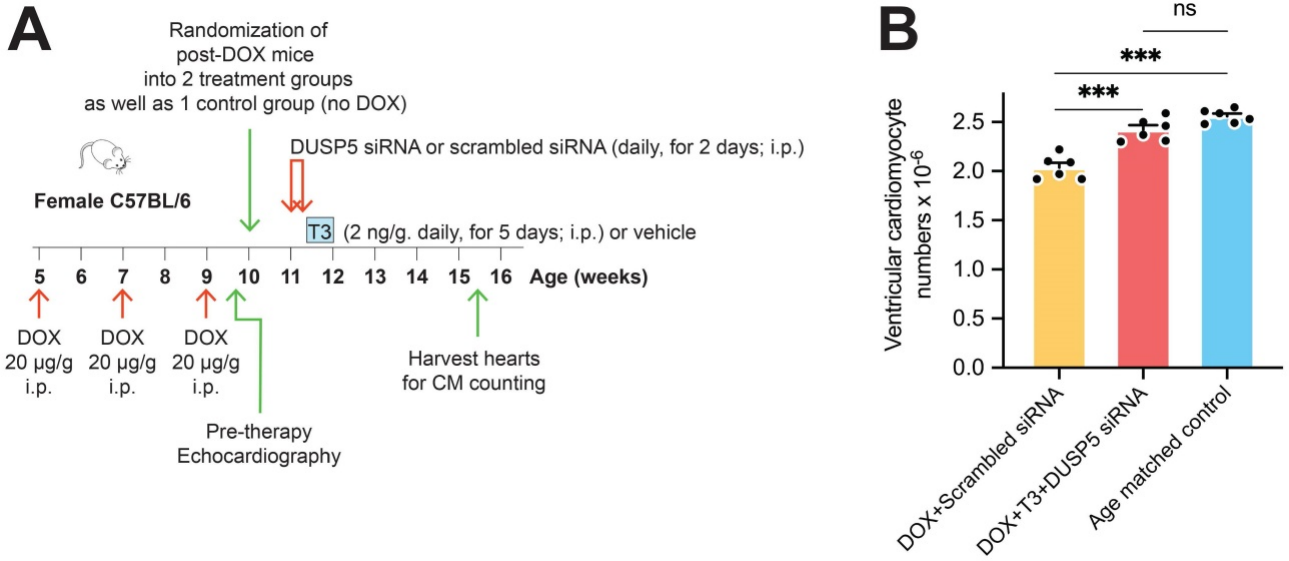
** Transient DUSP5 siRNA+T3 therapy increases cardiac muscle cell number in doxorubicin-injured hearts.** (A) Schematic showing the experimental protocol used to create doxorubicin-induced cardiotoxicity and implementation of transient therapies with scrambled siRNA (control) or DUSP5 siRNA+T3 therapy. (B) Total ventricular cardiomyocyte numbers in doxorubicin-treated mice were determined 3-weeks after scrambled siRNA or DUSP5 siRNA+T3 therapy as well as in untreated age-matched controls (*n* = 6-7 mice per group). Individual data points and mean ± s.e.m are shown. ***P < 0.001, ns: not significant. Comparisons between groups were made using ANOVA with Sidak multiple comparison test.

We first assessed the effect of DUSP5 siRNA+T3 therapy on ventricular cardiomyocyte numbers. Without DUSP5 siRNA+T3 therapy (that is, with just scrambled siRNA administration), doxorubicin-treated mice had ~20% fewer ventricular cardiomyocytes than age-matched controls (*P* < 0.001) (Figure 6A-B). By contrast, in doxorubicin-treated mice given DUSP5 siRNA+T3 therapy, cardiomyocyte numbers were 19% higher than in doxorubicin-treated mice given scrambled siRNA therapy (*P* < 0.001) (Figure 6B) but were not significantly different from age-matched controls (*P* = 0.068) (Figure 6B). These studies suggest that as in uninjured hearts (Figure 1C), DUSP5 siRNA+T3 therapy increases cardiomyocyte numbers in mice with preexisting doxorubicin injury.

Because, in cell culture studies, T3 reduces neonatal rat cardiomyocyte death from serum starvation by activating AKT 20, it is conceivable that T3, as a component of DUSP5 siRNA+T3 therapy, inhibits any progressive cardiomyocyte apoptosis that might be occurring long after the end of the final doxorubicin dose. However, we found that in hearts with preexisting doxorubicin injury, there was no difference in ventricular cardiomyocyte numbers with or without T3 therapy (2.02 ± 0.034 × 10^6^ and 2.03 ± 0.049 × 10^6^ cardiomyocytes in scrambled siRNA+T3-treated and scrambled siRNA mouse groups, respectively; *n* = 6/group; *P* = 0.87) or with or without DUSP5 siRNA therapy (2.08 ± 0.036 × 10^6^ and 2.03 ± 0.049 × 10^6^ cardiomyocytes in DUSP5 siRNA-treated and DUSP5 scrambled siRNA-treated animals, respectively; *n* = 6/group; *P* = 0.45). These findings argue against T3 or DUSP5 siRNA monotherapy having a cardioprotective effect in the setting of chronic doxorubicin injury.

Doxorubicin-induced decreases in cardiomyocyte numbers raised the possibility that LV muscle is lost as a result of doxorubicin toxicity; the finding that DUSP5 siRNA+T3 therapy increases cardiomyocyte number suggests that this therapy might restore LV muscle lost due to prior doxorubicin injury. To evaluate these possibilities, we performed transthoracic echocardiography soon after the last dose of doxorubicin and at 1- and 3-weeks after DUSP5 siRNA+T3 therapy.

**Figure 7:**
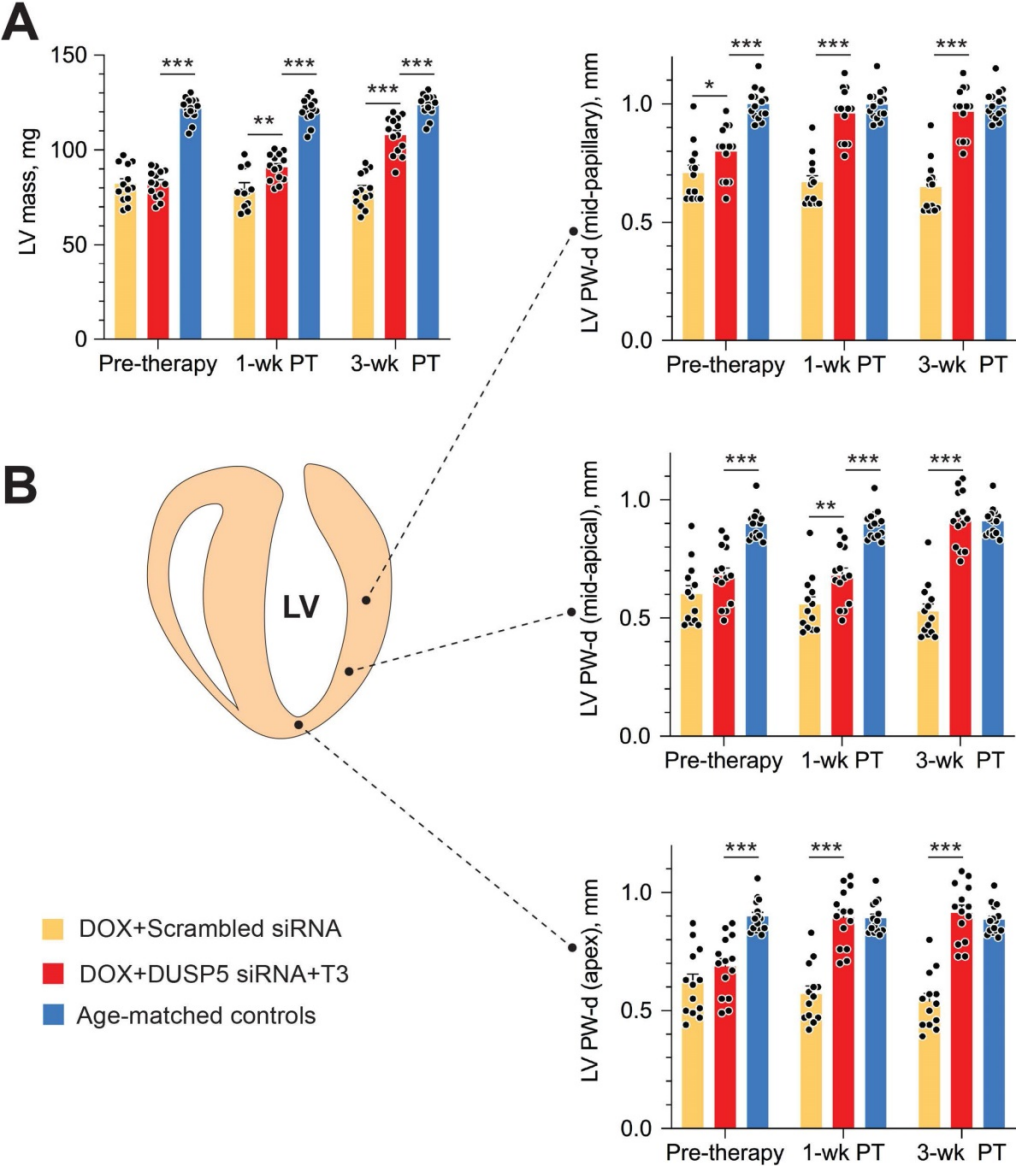
** Transient DUSP5 siRNA+T3 therapy rebuilds cardiac muscle in mice with doxorubicin cardiomyopathy.** (A-B) Repeated assessments in mice of LV mass (A) and posterior wall (PW) dimensions at end-diastole (B) at the LV mid-papillary level (top), mid-apical level (middle) and at the LV apex (bottom); assessments were made at the end of repeated doxorubicin exposure (pre-therapy) and 1- and 3-week after scrambled siRNA (control therapy) or DUSP5 siRNA+T3 therapy (*n* = 13-14 mice/group). Echocardiographic sampling sites are indicated in the diagram (middle left). Age-matched controls that were not exposed to doxorubicin or any therapy were also studied (*n* = 15). **P < 0.01, ***P < 0.001. Individual data points and mean ± s.e.m are shown. Comparisons with the doxorubicin+DUSP5 siRNA+T3 group were made using ANOVA with Sidak multiple comparison test. PT, post-therapy; DOX, doxorubicin.

Repeated doxorubicin treatment decreased LV mass by 32% (*P* < 0.0001) (Figure 7A), and also decreased PW thicknesses at the LV mid-papillary and mid-apical level as well as thickness of the LV apex by 25%-30% (*P* < 0.001 in each case) (Figure 7B). Thus, the main effects of repeated doxorubicin exposure were a loss of LV muscle as characterized by a decrease in LV mass and global LV PW thinning.

**Figure 8:**
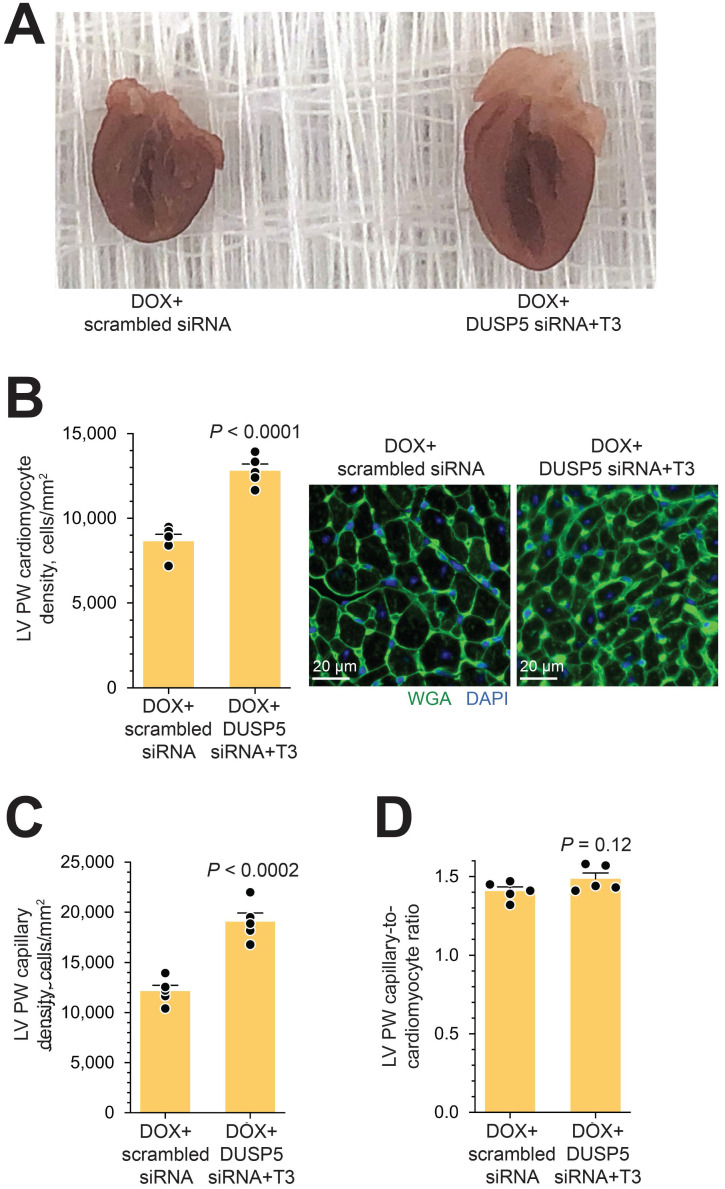
** Gross morphology and cellular composition of doxorubicin-injured LVs following parenteral DUSP5 siRNA+T3 therapy.** (A) Gross appearance of doxorubicin-injured hearts cut in the longitudinal plane studied at 3-weeks following DUSP5 siRNA+T3 or control DUSP5 scrambled siRNA therapy. (B-C) LV PW cardiomyocyte (B) and capillary densities (C) were assessed after staining with wheat-germ agglutinin (WGA) and 4′,6-diamidino-2-phenylindole (DAPI) to identify cell borders and nuclei, respectively. Representative images are shown in B (right). Bars, 20 µm. Determinations were made using tissue sections from the LV mid-papillary region. (D) Capillary-to cardiomyocyte ratio. Comparison between groups were made using a 2-tailed *t*-test (*n* = 5 mice per group in B-D). Individual data points and mean values ± s.e.m are shown.

In uninjured hearts, the increase in LV PW observed with DUSP5 siRNA+T3 therapy was not uniform, with major effects seen in the periapical LV myocardium (Figure 2B-D). But LV PW thinning after repeated doxorubicin exposure was global (Figure 7B). To determine if similar non-uniformity of LV muscle regeneration is also observed with DUSP5 siRNA+T3 treatment of doxorubicin-injured hearts, we determined PW thickness at multiple LV levels. This showed that after DUSP5 siRNA+T3 therapy, PW thickness was similar to that of age-matched controls at all levels throughout the LV, that is, at the LV mid-papillary and mid-apical levels and at the LV apex, whereas before the start of therapy they were all about 20%-33% thinner, respectively (*P* < 0.001) (Figure 7B). No such LV PW thickening was observed after scrambled siRNA therapy (Figure 7B). The gross appearance of the heart after DUSP5 siRNA+T3 or scrambled siRNA therapy is shown in Figure 8A. In the rebuilt LV myocardium of DUSP5 siRNA+T3-treated mice, cardiomyogenesis was apparent by the finding that LV PW cardiomyocyte and capillary densities were 48% and 57% higher, respectively, than in mice treated with scrambled siRNA (*P* < 0.0001 in both cases) (Figure 8B-C), while the capillary-to-cardiomyocyte ratio was maintained at ~1.4 (Figure 8D). Although there was a trend to a reduction in myocardial fibrosis with DUSP5 siRNA+T3 treatment, this failed to reach statistical significance (Figure S7). It is possible that a salutary effect on fibrosis might be observed with a longer period of follow-up.

**Figure 9:**
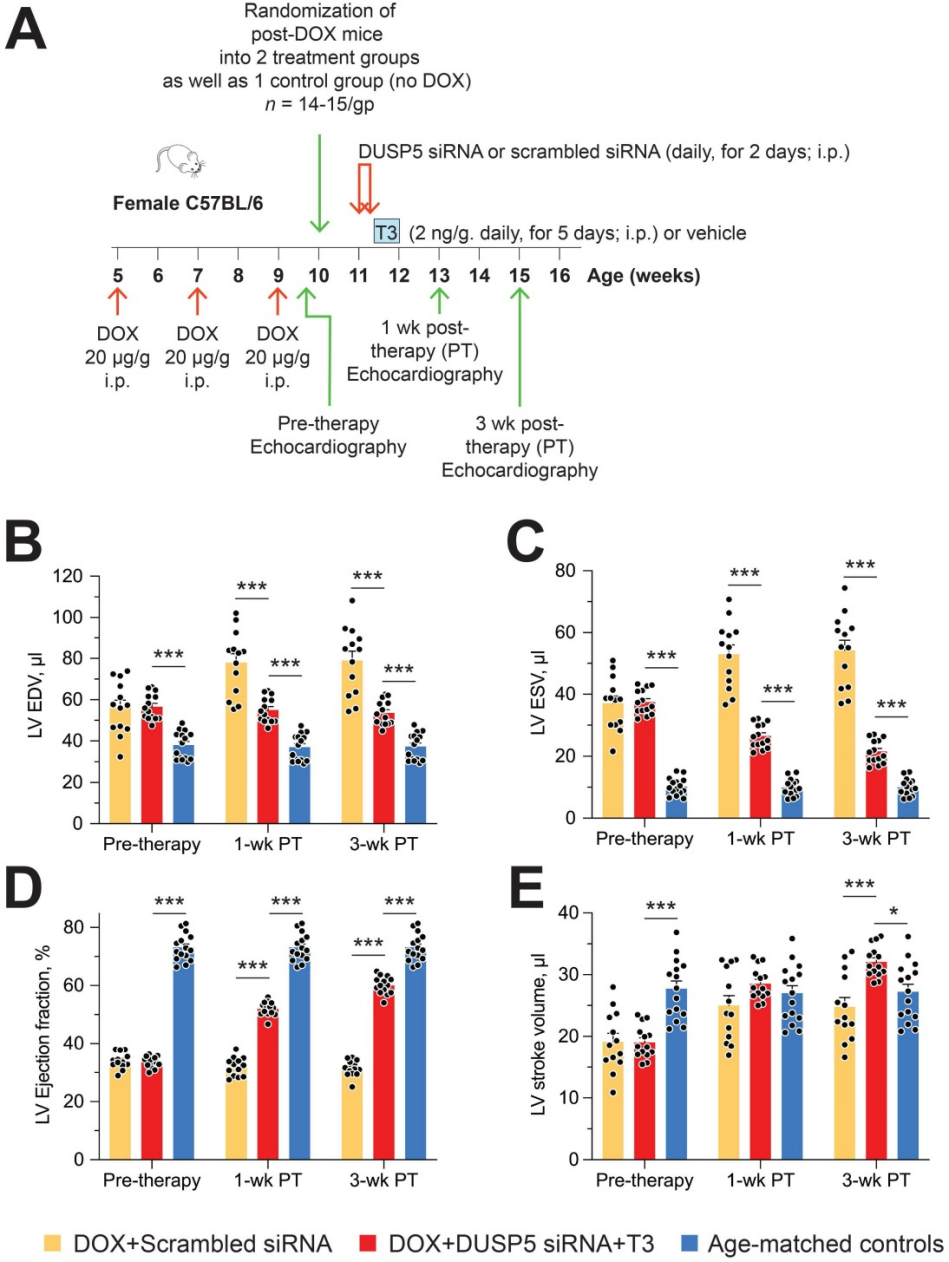
** Transient DUSP5 siRNA+T3 therapy reverses LV dysfunction in doxorubicin-injured hearts.** (A) Diagram showing the experimental protocol used to create doxorubicin-induced cardiotoxicity and implementation of transient therapies with DUSP5 scrambled siRNA (control) or DUSP5 siRNA+T3 therapy. (B-E) Repeated assessments in mice of LV end-diastolic volume (EDV) (B), end-systolic volume (ESV) (C), LV ejection fraction (D) and LV stroke volume (E) at the end of repeated doxorubicin exposure (pre-therapy) and 1- and 3-week after scrambled siRNA or DUSP5 siRNA+T3 therapy (*n* = 13-14 mice/group). These temporal assessments were also made in age-matched controls that were not exposed to doxorubicin or any therapy (*n* = 15). ***P < 0.0001. Individual data points and mean ± s.e.m are shown. Comparisons with the doxorubicin+DUSP5 siRNA+T3 group were made using ANOVA with Sidak multiple comparison test. PT, post-therapy; DOX, doxorubicin; CM, cardiomyocyte.

Restoration of LV muscle, together with improved LV contractile function, are important goals of regenerative therapy. We, therefore, studied effect of chronic doxorubicin injury as well as the effect of DUSP5 siRNA+T3 or control (scrambled siRNA) therapy on LV contractile function. Transthoracic echocardiography was performed 5 days after the last dose of doxorubicin and at 1- and 3-weeks after DUSP5 siRNA+T3 therapy (Figure 9A). We also studied untreated age-matched controls. Repeated doxorubicin exposure resulted in a 45% increase in EDV (Figure 9B) and a 3.6-fold increase in ESV (Figure 9C) (*P* < 0.001, in each case). These LV volume changes produced a ~40-percentage point decrease in ejection fraction (*P* < 0.0001) (Figure 9D) and a 32% decrease in stroke volume (*P* < 0.0001) (Figure 9E).

Treatment with control therapy (scrambled siRNA) starting at 5 days post-doxorubicin injury did not improve any of the above indices of LV contractile function over the 3-week follow-up period (Figure 9B-E). By contrast, DUSP5 siRNA+T3 therapy progressively decreased LV ESV (Figure 9C) suggesting improvement in LV contractile function that should be reflected in LV ejection fraction and stroke volume. As described above, before the start of DUSP5 siRNA+T3 therapy, LV ejection fraction was ~40-percentage point lower than that of age-matched controls (Figure 9D) and stroke volume was 32% lower (Figure 9E) (*P* < 0.0001, in each case), whereas 3-weeks after therapy, they were only ~13-percentage points lower and 12% higher than age-matched controls, respectively (*P* < 0.0001, in each case) (Figure 9D-E), indicating substantial restoration of LV contractile function.

LV dilatation is an important aspect of adverse LV remodeling. After repeated doxorubicin treatments, LV chambers were about 1.45-fold larger than those of age-matched controls (*P* < 0.0001) (Figure 9B). Following scrambled siRNA therapy, they continued to enlarge, such that by 3-weeks post-therapy they were 2.1-fold larger (*P* < 0.0001) (Figure 9B). Extant LV dilatation was not reversed by DUSP5 siRNA+T3 therapy, but it completely prevented the progressive chamber dilatation, evident from increases in EDVs, that was observed in animals treated with scrambled siRNA (Figure 9B).

**Figure 10:**
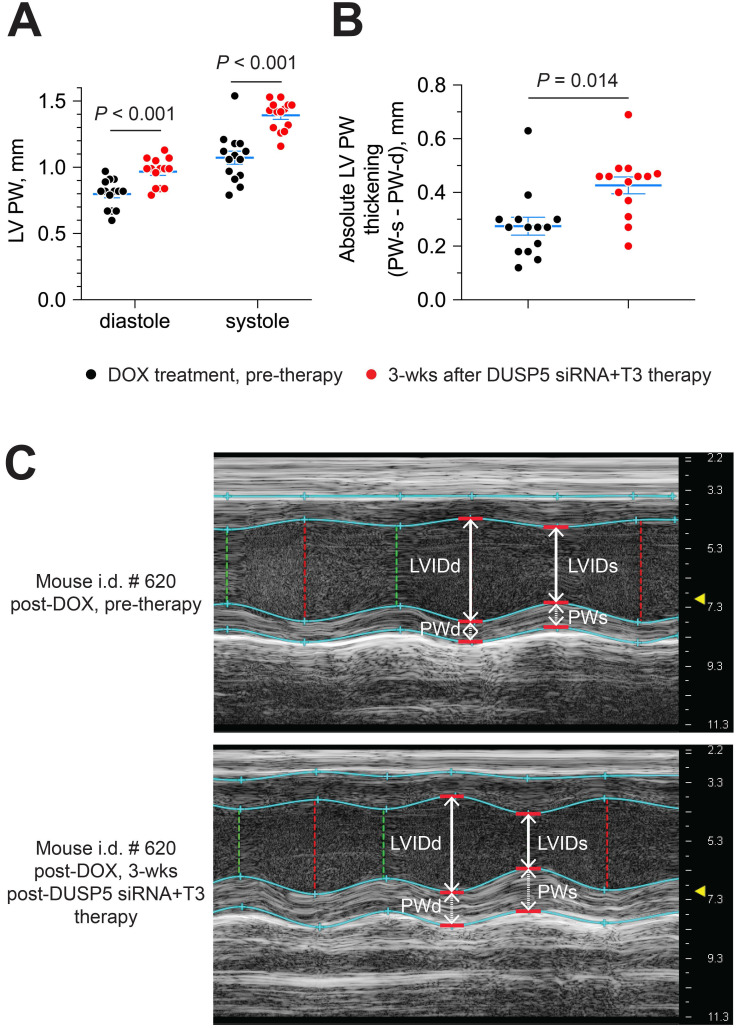
** Parenteral DUSP5 siRNA+T3 therapy potentiates contractile function at the LV mid-papillary level in mice with doxorubicin cardiomyopathy.** (A-B) LV posterior wall (PW) dimensions at diastole and systole (A), and absolute LV PW thickening at systole (B), before and 3 weeks after DUSP5 siRNA+T3 therapy in doxorubicin-injured hearts (*n* = 14 mice per group). Comparisons were made using a 2-tailed *t*-test. Individual data points and mean ± s.e.m are shown. (C) Representative M-mode images of a mouse heart before and after DUSP5 siRNA+T3 therapy. LV internal dimensions at diastole (LVIDd) and systole (LVIDs) as well as PW dimensions at diastole (PWd) and systole (PWs) are indicated.

Next, we evaluated if DUSP5 siRNA+T3 therapy enhances LV PW contractility. For this purpose, we compared LV PW dimensions at systole and diastole, before and then 3 weeks after DUSP5 siRNA+T3 therapy. LV PW dimensions (assessed at the mid-papillary level) at end-diastole and end-systole increased by 22% and 30% (*P* < 0.001, in each case), respectively, after DUSP5 siRNA+T3 therapy (Figure 10A). As a result, systole-associated absolute LV PW thickening (LV PWs - LV PWd) (Figure 10B), which is a measure of localized LV PW contractility, increased by 54% (*P* = 0.014).

Long-term, doxorubicin-treated mice given acute DUSP5 siRNA+T3 therapy generally looked well: they were not lethargic, had normal posture and all 14 mice survived over the 3-week post-therapy follow-up. By comparison, doxorubicin-treated mice given scrambled siRNA therapy were in poor health. All 14 animals were lethargic or immobile, had a hunched posture, were poorly groomed and clearly runted (Figure S8), and one died in the post-therapy period. In addition, 3-weeks post-therapy, doxorubicin-treated mice given scrambled siRNA therapy had lower body weights than those given DUSP5 siRNA+T3 therapy (body weights were 16.6 ± 0.63 g and 18.5 ± 0.45 g for mice given scrambled siRNA and DUSP5 siRNA+T3 therapy, respectively; *P* < 0.05). Because our predetermined criteria for signs of pain included lack of grooming, sitting in a hunched position, abdominal breathing and body weight loss, these scrambled siRNA-treated mice had to be euthanized at 3-weeks post therapy.

## Discussion and Conclusions

The evidence presented here suggests that brief (7-day) therapy with DUSP5 siRNA+T3 builds LV muscle in uninjured hearts and rebuilds LV muscle in chronic doxorubicin-injured hearts. In uninjured hearts, DUSP5 siRNA+T3 therapy increased cardiomyocyte endowment of the heart, enhancing LV thickness and contractile function; effects that were sustained for up to 4-weeks post-therapy. Danusertib, an inhibitor of mitosis 18, prevented these salutary effects of DUSP5 siRNA+T3 therapy, which suggests that an expanded LV myocardium with improved contractile function requires increases in cardiomyocyte numbers. Thus, short term DUSP5 siRNA+T3 treatment is potentially a viable therapeutic approach for treating doxorubicin cardiomyopathy, a condition for which no specific therapy is currently available 21. As an acute pharmacological approach, it carries the additional benefit of potentially being rapidly translatable into an effective therapeutic strategy for the management of patients with doxorubicin cardiomyopathy.

We demonstrate that transient therapy with DUSP5 siRNA+T3 therapy rebuilds heart muscle in ~12-week-old adult mice with chronic doxorubicin cardiomyopathy through addition of cardiac muscle cells (Figures 6B and 8B) and neovascularization (Figure 8C); processes that, within a few weeks after the therapy, normalize LV contractile function (Fig. 9C-E and Figure 10A-C). Taken together, our data show that the limited regenerative potential of adult hearts can be therapeutically enhanced.

ERK1/2 phosphorylation and translocation of the phosphoprotein from the cytosol to the nucleus connects growth factor signaling with transcriptional activation of cell cycle promoting genes and cell proliferation 22,23. In neonatal P2 cardiomyocytes, ERK1/2 activation is required for T3-stimulated cardiomyocyte proliferation 10. As LV cardiomyocytes mature, the expression of DUSP5, a nuclear phosphatase that inhibits p-ERK1/2, increases, starting immediately after the neonatal period (which ends at P6) 11. We show here that, in young adult mice, DUSP5 knockdown in LV cardiomyocytes using DUSP5 siRNA amplifies T3-stimulated increases in p-ERK1/2 and cyclin D1 and B1 and increases ventricular cardiomyocyte numbers.

With respect to cell proliferation, are mature cardiomyocytes the primary targets of T3 and DUSP5 siRNA therapy. Our findings in neonatal murine hearts suggest an autocrine mechanism involving IGF-1 10. T3 increases IGF-1 expression in primary cardiomyocytes in culture as well as ERK1/2 proliferative signaling, which is inhibited by IGF-1 depletion using IGF-1 siRNA 10. These T3 effects are seen in serum-free media, but not in media containing serum. Presumably, this is because the T3 proliferative effect is mediated by growth factor secretion (i.e., IGF-1), the effects of which are likely to be masked by the abundance of growth factors present in serum. Because adult cardiomyocytes, unlike their neonatal counterparts, require serum-containing media for viability (data not shown), we were unable to provide *in vitro* evidence that IGF-1 stimulation by T3 is also limited to cardiomyocytes in adult. However, a number of findings support the notion that T3 and DUSP5 siRNA directly target cardiomyocytes. First, we show that T3 increases IGF-1 expression in isolated cardiomyocytes from neonatal hearts but not in cardiac non-myocytes, which prominently include cardiac fibroblasts. Second, *in vivo*, T3 increases IGF-1 expression in adult cardiomyocytes, but not in non-myocytes. Third, DUSP5 is expressed in adult cardiomyocytes, but not in cardiac non-myocytes 11. Together, these findings suggest that cardiomyocytes are an important direct target of T3 and DUSP5 siRNA therapy in the adult heart. However, these findings cannot rule out the possibility that some of the beneficial effects of T3+DUSP5 siRNA therapy on the heart might be indirect, being derived from stimulation of effectors, such as growth factors, from other organs. As such, this uncertainty represents a mechanistic limitation of our study.

Identification of cardiomyocyte proliferation *in vivo* is challenging. While determination of S-phase and mitosis in these cells is possible using, for example, EdU and pH3 labeling, respectively, it leaves open the possibility that the effects of mitogenic signaling terminate in increased nuclear ploidy, multinucleation or cell replication. Also, even if cardiomyocytes display aurora B or mitotic kinesin-like protein-1 labeling at their mid-body, the identification of cleavage furrows in tissue sections as a measure of cell replication, is subjective. Use of genetic lineage tracing to demonstrate cardiomyocyte replication overcomes some of these concerns. Previously, a multicolor fluorescent reporter was used to provide an independent method to measure productive, mitogen-stimulated cardiomyocyte proliferation 11,24. In double-transgenic *Myh6*-Mer*Cre*Mer::Rosa26fs-Confetti mice, we used minimal 4-hydroxytamoxifen-induced Cre expression in cardiomyocytes to produce monochromatic labeling in < 2% of cardiomyocytes. DUSP5 siRNA+T3 therapy resulted in a twofold increase in monochromatic clusters over that seen in the absence of mitogenic signaling, providing evidence of *in vivo* cardiomyocyte replication. However, genetic lineage tracing has the potential to grossly underestimate the “true” increase in cardiomyocyte replication-events driven by DUSP5 siRNA+T3 therapy because this technique demands minimal cardiomyocytes labeling to reduce baseline noise. As a result, only a small subset (< 2%) of LV cardiomyocytes can be evaluated for replication. While this provides robust evidence that DUSP5 siRNA+T3 therapy induces cardiomyocyte replication, it does not allow the extent of replication to be determined.

To overcome this limitation, we used direct cell counting, which is widely used to determine the extent of cardiac muscle cell expansion 8,25-27. Incomplete tissue digestion is a limitation of the direct cell counting method 28. We therefore optimized enzymatic heart disaggregation 10-11 to ensure that digestion efficiencies exceeded 97%. This allowed us to show that DUSP5 siRNA+T3 therapy to mice with uninjured hearts increases cardiomyocyte numbers by ~25% (*P* < 0.001); an effect that could be blocked by cell cycle inhibition. Moreover, in support of the notion that DUSP5 siRNA+T3 drives cardiomyocyte proliferation, we found that this therapy increased the weight of the ventricular myocardium by 15%; an effect not due to hypertrophy, given that cardiomyocytes size fell by 10-15%.

T3 effects are dependent on dose and duration of therapy. At high doses, exogenous T3 administration (e.g., 15-50 ng/g), when sustained over long periods (e.g., for 21 days), results in hyperthermia, body weight loss and increased heart weight 29. We administered T3, 2 ng/g/day, over 5 days as a component of the cardiac regeneration protocol. In the absence of DUSP5 siRNA treatment, this dose/duration of T3 therapy did not impact heart or body weight and did not increase body temperature. Also, we found no evidence of cardiac arrhythmias in dozens of echocardiographic analyses performed on T3-treated mice with or without DUSP5 siRNA therapy.

A key question is how intraperitoneal administration of the DUSP5 siRNA molecule can result in target specificity. T3 activates ERK1/2 proliferative signaling in cardiomyocytes secondary to increased *Igf1* and *Igf1r* expression, both of which are required for this T3 action 10. Although *Igf1* has a validated thyroid responsive element (TRE) in its promoter 30, T3 stimulates *Igf1* transcription in cardiomyocytes through a redox mechanism that activates a rare c-Jun N-terminal kinase (JNK) isoform, JNK2α2 10. The molecular mechanism by which T3 activates *Igf1r* transcription in cardiomyocytes is uncertain. Molecular heterogeneity in signaling mechanisms between cells is likely to dictate which cells proliferate in response to T3. An examination of organ and body weights revealed that low-dose T3, in combination with DUSP5 siRNA therapy, selectively increased LV growth, but not that of other heart chambers or of many other organs examined.

RNA-based drugs have recently emerged to show great therapeutic potential in treating diverse human diseases which are otherwise undruggable due to lack of small molecules 31-33. We used polyethylenimine-derived *in vivo*-jetPEI transfection reagent to deliver siRNA into the mouse; it is also an effective means of delivering DNA and RNA in humans 34,35, which potentially makes DUSP5 siRNA+T3 therapy easily translatable. Doxorubicin is one of the most important antineoplastic drugs but, repeated use, as in cancer therapy, amplifies its cardiotoxicity, thereby limiting its antitumor efficacy 21. Given the findings here, transient DUSP5 siRNA+T3 therapy may prove useful for sustained restoration of LV muscle and contractile function in patients with doxorubicin cardiomyopathy. Moreover, the specificity of the DUSP5 siRNA + T3 proliferative effects for cardiomyocytes, as also evident by the absence of growth of other organs, such as liver, lungs or kidneys (Table 1), as well as the brief (5 day) period of therapy required to stimulate cardiomyocyte replication, suggests that this therapy will be safe and not stimulate neoplastic cell growth, even in patients with residual tumor. It is noteworthy that T3 signaling, through its canonical nuclear receptor, actually suppresses tumor growth in certain cases 36-38. However, in future studies, these possibilities will need to be confirmed in the setting of T3+DUSP5 siRNA therapy.

For preclinical development of regenerative therapy, doxorubicin-induced cardiomyopathy in mice represents an appropriate small animal model that replicates some of the important cytotoxic effects of this cancer therapeutic in the human heart. Therapeutic assessment of T3+DUSP5 siRNA treatment, in this disease model, has been aided by technological advances in echocardiography, which allow a detailed assessment of the therapy on cardiac morphology and contractile function. However, while the mouse model provides rapid and economical testing and adequate group sizes to ensure sufficient statistical power, it has disadvantages. Cardiac repair studies show larger effects in rodents than in large animal studies; responses in the latter relating better to clinical trials in humans 39. Such considerations dictate that the studies described here be replicated in large animal models before translation into humans.

## Supplementary Material

Supplementary figures and video legends.

Supplementary movie 1.

Supplementary movie 2.

## Figures and Tables

**Table 1:** Body and organ weights and core body temperatures of 5-week-old mice treated with DUSP5 siRNA or scrambled siRNA, with or without T3 therapy

	DUSP5 scrambled siRNA^a^		DUSP5 siRNA^a^	
	Vehicle	T3	Vehicle	T3
Body weight, g	21.8 ± 0.18	21.8 ± 0.15^N.S.^	21.8 ± 0.2	21.7 ± 0.15^N.S.^
Atria/body weight, mg/g	1.27 ± 0.015	1.25 ± 0.015^N.S.^	1.27 ± 0.01	1.26 ± 0.05^N.S.^
Ventricle/body weight, mg/g	4.98 ± 0.03	4.98 ± 0.03^N.S.^	4.97 ± 0.04	5.71 ± 0.1***
RV/body weight, mg/g	1.67 ± 0.025	1.67 ± 0.02^N.S.^	1.67 ± 0.019	1.60 ± 0.076^N.S.^
LV/body weight, mg/g	3.31 ± 0.036	3.3 ± 0.014^N.S.^	3.3 ± 0.027	4.11 ± 0.04***
Lung/body weight, mg/g	7.23 ± 0.036	7.25 ± 0.012^N.S.^	7.24 ± 0.024	7.25 ± 0.018^N.S.^
Kidneys/body weight, mg/g	21.4 ± 0.82	21.8 ± 0.43^N.S.^	21.6 ± 0.75	21.7 ± 0.69^N.S.^
Liver/body weight, mg/g	50.5 ± 0.32	50.5 ± 0.14^N.S.^	50.5 ± 0.37	50.8 ± 0.36^N.S.^
Core body temperature, ºC	37.1 ± 0.14	37 ± 0.31^N.S.^	37 ± 0.28	37 ± 0.23^N.S.^
*n*	6	6	6	6

^a^Five-week-old male C57BL/6 mice were given DUSP5 siRNA or control scrambled siRNA therapy daily for 2 days. T3 (2 ng/g, i.p., daily) or vehicle was then administered for 5 days starting on the day after the cessation of siRNA therapy. Core body temperatures were assessed at 2 weeks after cessation of T3 therapy. Immediately after this measurement, mice were euthanized and body and organ weights determined. Values are mean ± s.e.m. Within group (DUSP5 siRNA-treated or scrambled siRNA-treated mice) comparisons between vehicle versus T3 treated mice were made using a 2-tailed *t*-test. ***P < 0.001, N.S. (not significant) versus respective vehicle treatments.

## Abbreviations

CFP: cyan fluorescent protein
CM: cardiomyocyte
DOX: doxorubicin
DUSP5: dual specificity phosphatase 5
EDV: LV volumes at end-diastole
ESV: LV volumes at end-systole
GFP: green fluorescent protein
EF: ejection fraction
IACUC: Animal Care and Use Committee
IGF-1: insulin-like growth factor 1
IGF-1R: IGF-1 receptor
IVS-d: intraventricular septum at diastole
LV: left ventricle
LVID-d: LV internal dimensions at diastole
LVPW-d: LV posterior wall thickness at diastole
Myh6: myosin heavy chain-6
PW: posterior wall
RFP: red fluorescent protein
SV: stroke volume
T3: thyroid hormone
WGA: wheat germ agglutinin
YFP: yellow fluorescent protein
