# Supplementary material for: Thyroid hormone plus dual-specificity phosphatase-5 siRNA increases the number of cardiac muscle cells and improves left ventricular contractile function in chronic doxorubicin-injured hearts
Source: Theranostics. 2021 Mar 4;11(10):4790–808. doi: 10.7150/thno.57456 (PMC7978295; doi:10.7150/thno.57456)
Supplement: Supplementary file 1 — Supplementary figures and video legends. [file thnov11p4790s1.pdf]

Supplementary Material

Thyroid hormone plus dual-specificity phosphatase-5 siRNA increases the number of cardiac muscle cells and improves left ventricular contractile function in chronic doxorubicin-injured hearts

Lin Tan[1,#], Nikolay Bogush[1,#], Emmen Naqvi[1], John W. Calvert[2], Robert M. Graham[3], W. Robert Taylor[1,4,5], Nawazish Naqvi[1,*] and Ahsan Husain[1,*]

1. Department of Medicine (Cardiology), Emory University School of Medicine, Atlanta, GA, USA.
2. Department of Surgery, Carlyle Fraser Heart Center, Emory University School of Medicine, Atlanta, GA, USA.
3. Victor Chang Cardiac Research Institute, Darlinghurst, New South Wales, Australia.
4. Atlanta Veterans Affairs Medical Center, Cardiology Division, Atlanta, GA, USA.
5. Emory University School of Medicine and Georgia Institute of Technology, Department of Biomedical Engineering, Atlanta, GA, USA.

# These authors contributed equally: Lin Tan, Nikolay Bogush.
* Corresponding authors: Ahsan Husain, Phone: 404-727 8125, E-mail: ahusai2@emory.edu or Nawazish Naqvi, Phone: 205-253 1190, E-mail: nnaqvi@emory.edu

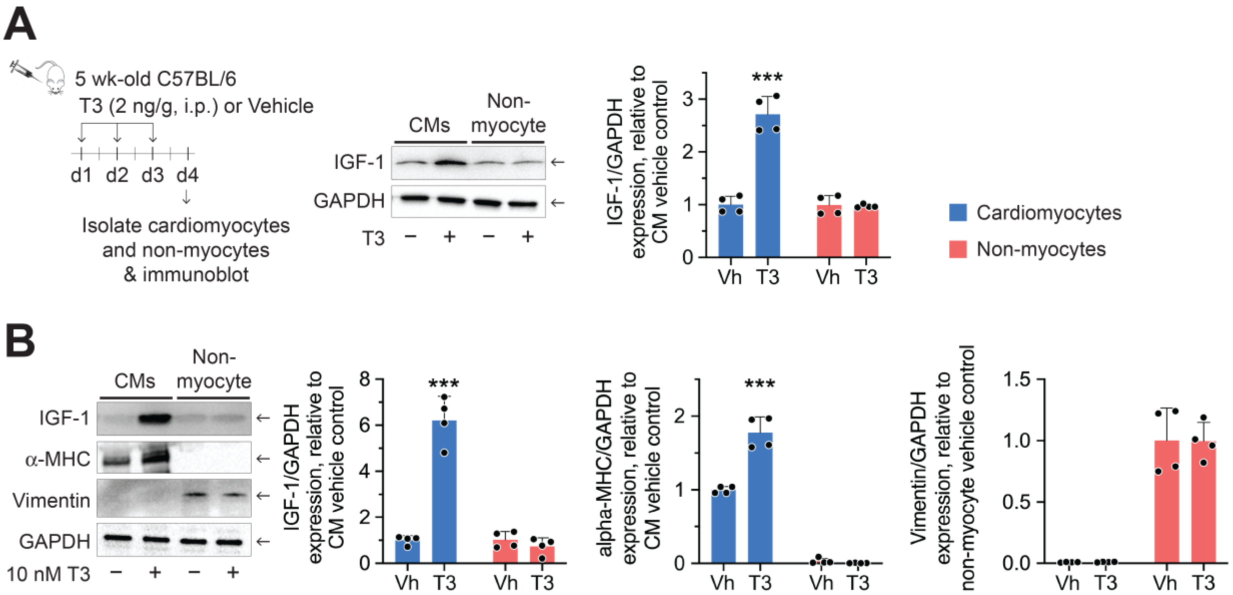

Figure S1. T3-stimulated IGF-1 expression is restricted to left ventricular cardiomyocytes. (A) Schematic (left) illustrates experimental protocol for *in vivo* study in mice showing dose and duration of T3 administration and timing of harvesting hearts for isolation of LV cardiomyocytes and non-myocytes for immunoblot analyses. Representative immunoblots show indicated proteins expressed in cardiomyocytes (CMs) and non-myocytes in T3 (+) or vehicle (–) injected mice. Bar graph (right) shows quantification of the IGF-1 expression in each experimental group (n = 4 mice per group). (B) Representative immunoblots show expression of indicated proteins in lysates prepared from neonatal CM and non-myocyte cultures treated with (+) or without (–) 10 nM T3. Bar graphs show quantitative analysis of IGF-1, alpha (α)-MHC and Vimentin (n = 4 independent biological replicates). Vh, vehicle. ***$P < 0.001$. Individual data points and mean ± s.e.m are shown. Comparisons were made using ANOVA with Sidak multiple comparison test.

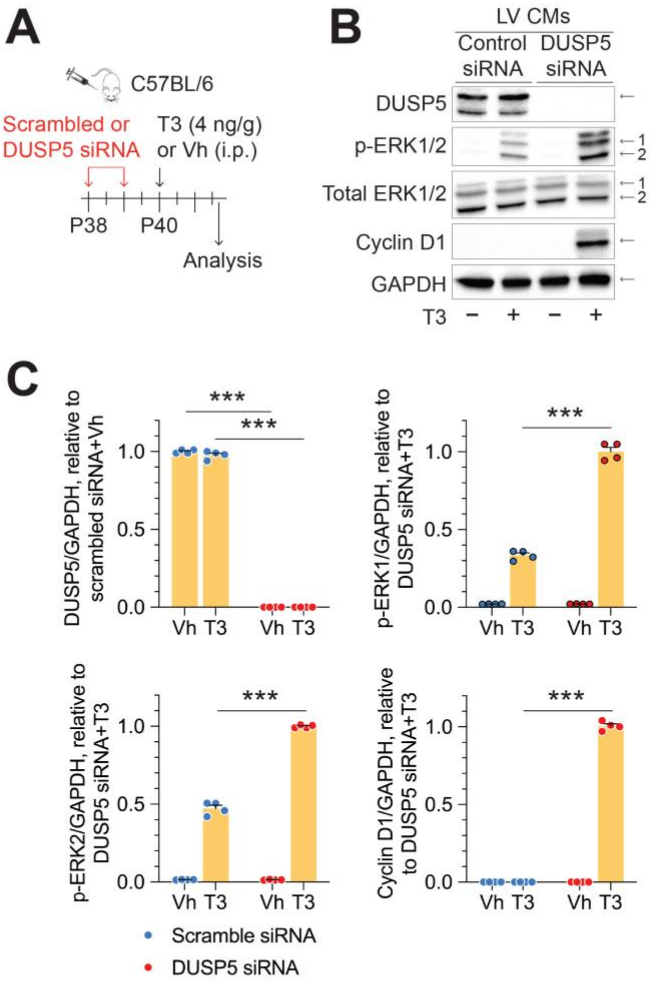

Figure S2. Acute DUSP5 siRNA+T3 therapy stimulates proliferative p-ERK1/2 signaling in adult cardiomyocytes *in vivo*. (A) The schematic shows experimental protocol. (B) Representative examples of immunoblots for the indicated proteins expressed in LV cardiomyocytes (CMs). (C) Bar graphs showing quantitative analysis of DUSP5 (*upper left panel*), p-ERK1 (*upper right panel*), p-ERK2 (*lower left panel*) and cyclin D1 (*lower right panel*) in LV CM lysates of 5-week-old mice treated with or without DUSP5 siRNA and T3 (n = 4 independent biological replicates); Vh, vehicle. ***$P < 0.001$. Individual data points and mean ± s.e.m are shown. Comparisons were made using ANOVA with Sidak multiple comparison test.

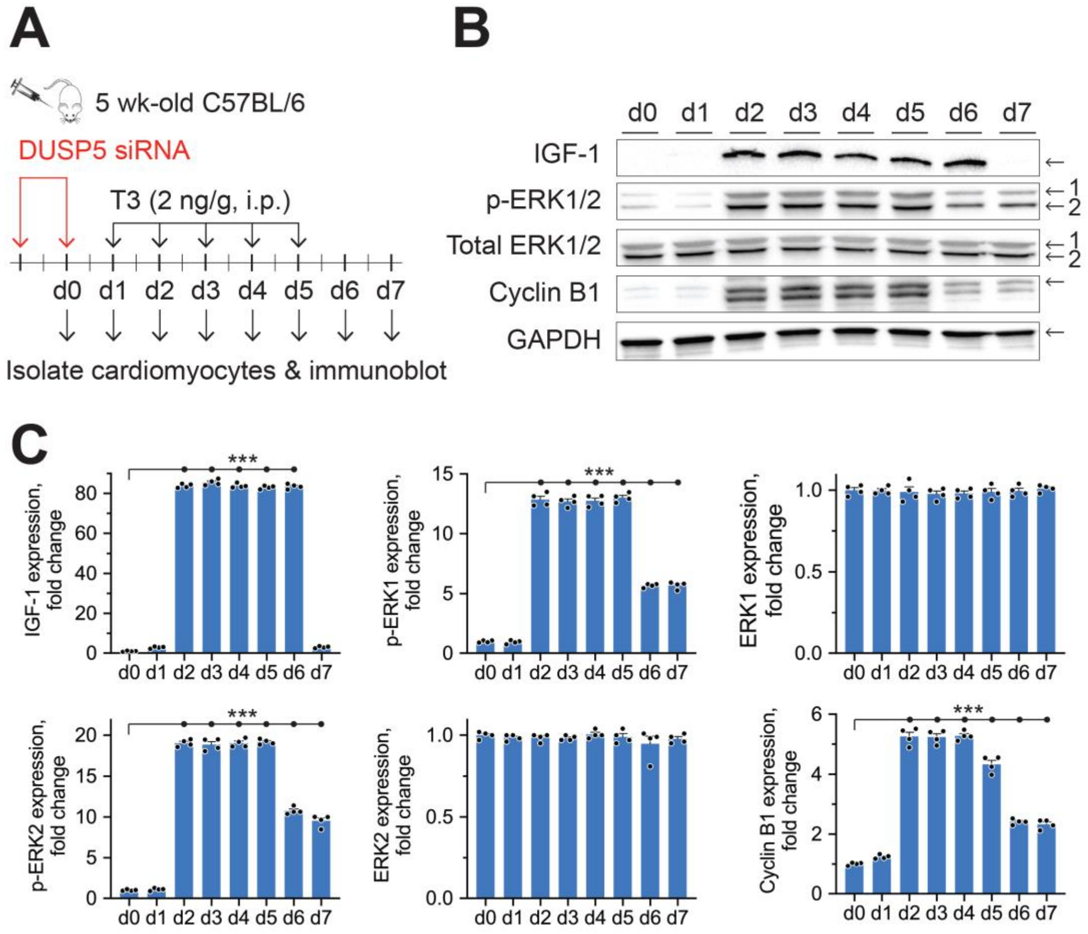

Figure S3. Time course of proliferative IGF-1/ERK1/2 signaling in adult LV cardiomyocytes post *in vivo* DUSP5 siRNA+T3 therapy. (A) The schematic shows experimental protocol for DUSP5 siRNA and T3 administration. Hearts were collected from day (d)0 to d7 for preparation of LV cardiomyocyte lysates for immunoblot analyses. (B) Representative examples of immunoblots for the indicated proteins expressed in LV cardiomyocytes of 5-week-old mice treated with DUSP5 siRNA and T3 at different timepoints. (C) Bar graphs showing quantitative analysis of IGF-1, p-ERK1, ERK1, p-ERK2, ERK2 and cyclin B1 (n = 4 mice per group); ***$P < 0.001$. Individual data points and mean ± s.e.m are shown. Comparisons were made using ANOVA with Sidak multiple comparison test.

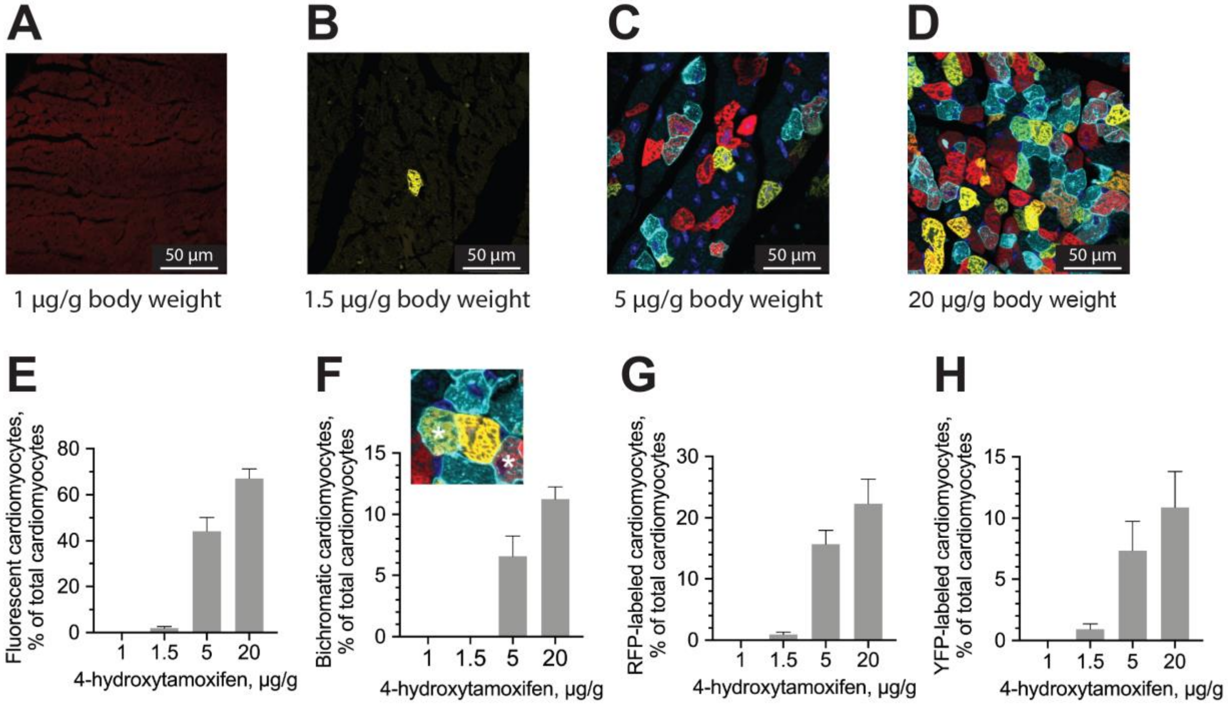

Figure S4. 4-Hydroxytamoxifen dose titration to achieve minimal fluorescent labeling of cardiomyocytes using scrambled siRNA+T3 treated *Myh6*-MerCreMer:: Rosa26-confetti mice. (A–D) Images show LV sections of mice treated with scrambled siRNA+T3 treated *Myh6*-MerCreMer:: Rosa26-confetti mice administered either 1, 1.5, 5 or 20 µg/g 4-hydroxytamoxifen. (E) Fluorescently labeled cardiomyocytes as a percentage of total cardiomyocytes in the four dose groups shown in *A-D*. (F) Bichromatic cardiomyocytes as a percentage of total cardiomyocytes–where each cardiomyocyte is simultaneously expressing two colors (indicated in the *inset* above the bar graph by asterisks). This could occur if both nuclei in a binuclear cardiomyocyte recombined randomly. Note: bichromatic cardiomyocytes were only seen with 4-hydroxytamoxifen doses >1.5 µg/g. (G-H) Frequency of red (RFP; G) and yellow (YFP; H) fluorescent protein-labeled cardiomyocytes as a percentage of total cardiomyocytes. Results shown are mean ± s.e.m.

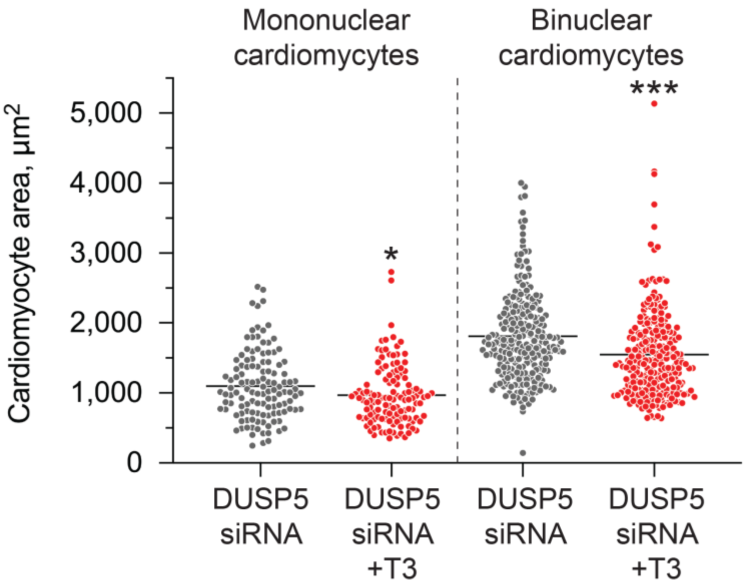

Figure S5. DUSP5 siRNA+T3 therapy given to 5-week-old mice results in a decrease in average ventricular cardiomyocyte size. Mononucleated (left) and binucleated cardiomyocyte (right) areas were smaller 4-weeks after DUSP5 siRNA+T3 therapy than after DUSP5 siRNA monotherapy. Ventricular cardiomyocyte areas were evaluated using Nikon NIS-Elements software; cardiomyocytes, in cell smears, were identified using cardiac troponin T-staining and nucleation state of the cell was determined by 4′,6-diamidino-2-phenylindole (DAPI) staining (n = 200 binucleated and 100 mononucleated cardiomyocytes pooled from the analysis of groups of 4 mice per therapy). Bars indicate mean values. Comparisons between the control (DUSP5 siRNA) and DUSP5 siRNA + T3 groups were made using a 2-tailed t-test. *$P < 0.05$, ***$P < 0.001$.

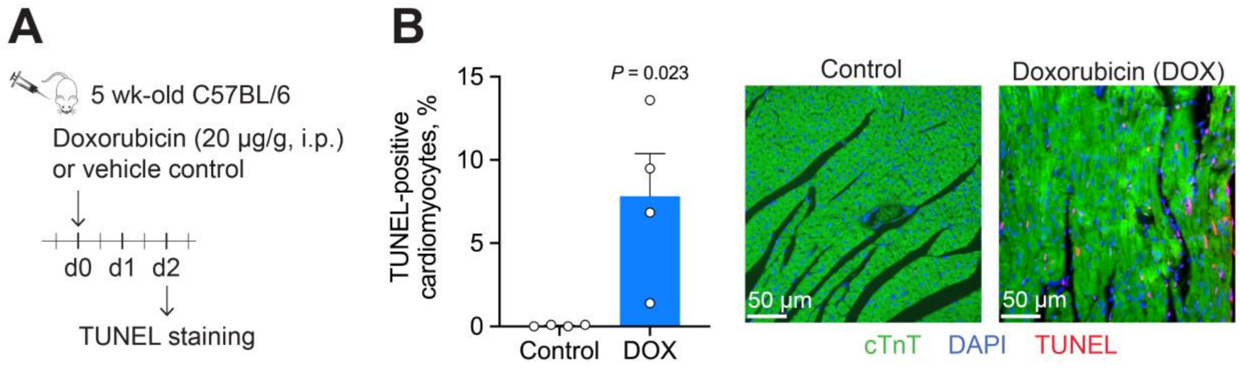

Figure S6. Acute doxorubicin cardiotoxicity induces marked apoptosis in cardiac muscle cells. (A) Schematic shows protocol for doxorubicin administration and timing for the harvesting of hearts for apoptosis analysis by TUNEL staining. (B) Representative images (right) show LV sections stained with cardiomyocyte specific marker cTnT (green) and TUNEL stain (red) from an uninjured (vehicle treated control) or doxorubicin (DOX)-treated mouse heart. Bar graph (left) shows quantification of the TUNEL positive apoptotic cardiomyocytes. Individual data points and mean ± s.e.m are shown. Statistical difference between the DOX-treated and control groups was determined using a 2-tailed *t*-test.

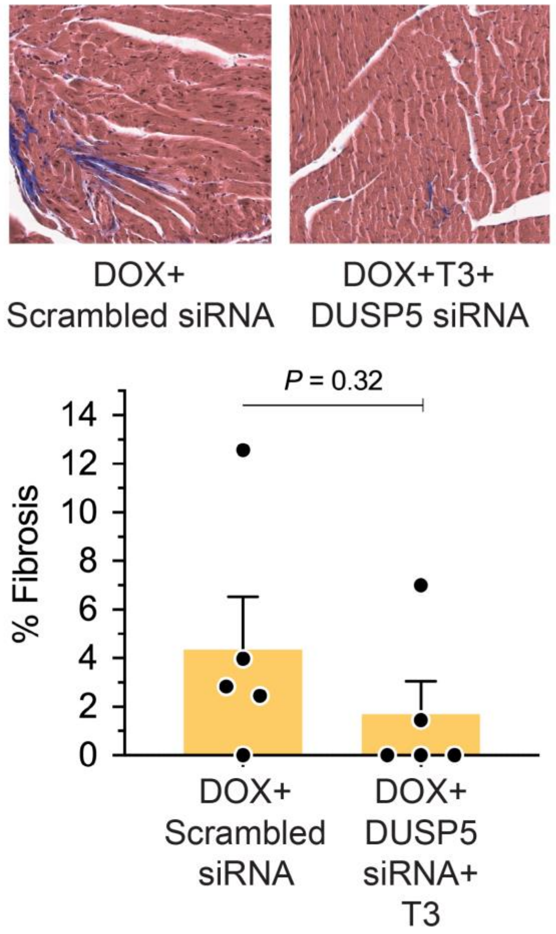

Figure S7. DUSP5 siRNA+T3 therapy did not significantly reduce fibrosis in doxorubicin (DOX)-treated mice. LV sections stained with Masson's trichrome showing fibrosis, which is stained blue, whereas cardiac muscle is stained red color. Bar graph below shows the quantification of interstitial fibrosis in mice treated with DUSP5 siRNA+T3 therapy and DUSP5 scrambled siRNA. Individual data points and mean ± s.e.m are shown. The *P* value for the comparison between the two groups was determined using a 2-tailed *t-test*.

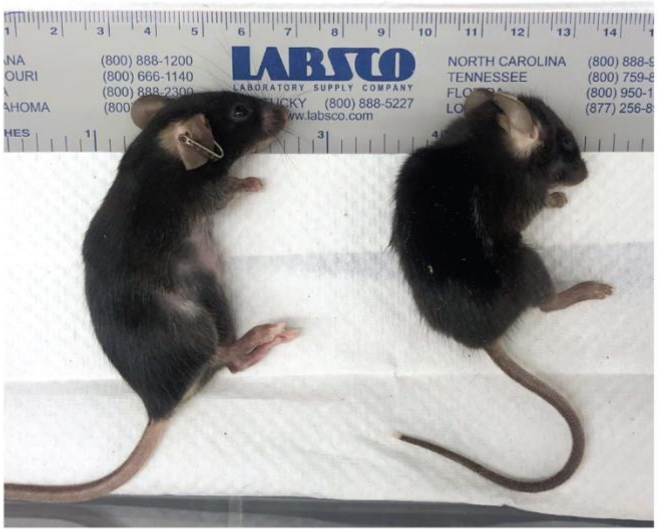

DOX+DUSP5 siRNA+T3 DOX+scrambled siRNA

Figure S8. Representative image of mice with doxorubicin cardiomyopathy 3 weeks after DUSP5 siRNA+T3 or scrambled siRNA (control) therapy. As is evident form the image, after scrambled siRNA (right), but not DUSP5 siRNA+T3 therapy (left) mice adopted a hunch-back posture and were runted.

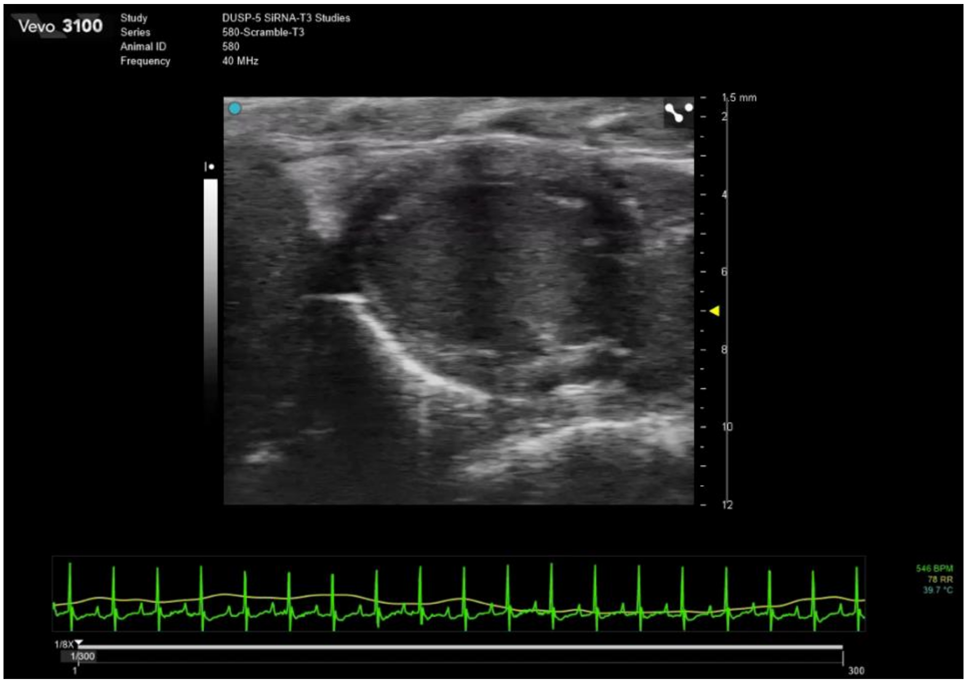

Video S1. Representative video showing the long-axis B-mode image of the contracting LV of a 5-week old mouse treated with DUSP5 scrambled siRNA+T3.

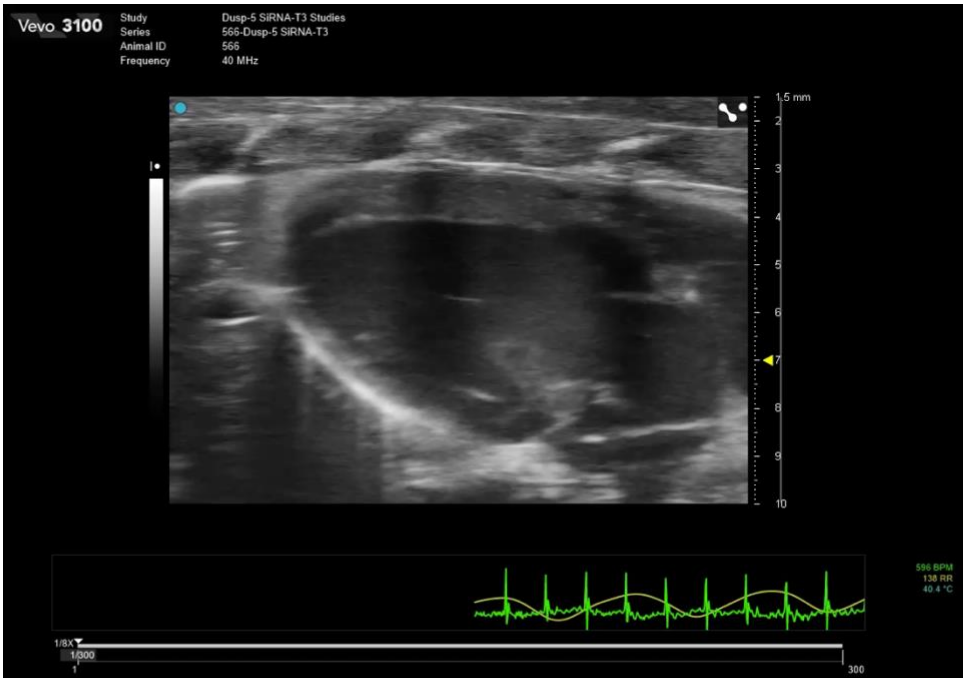

Video S2. Representative video showing the long-axis B-mode image of the contracting LV of a 5-week old mouse treated with DUSP5 siRNA+T3.
